# Compounds Derived from 9,9‐Dialkylfluorenes: Syntheses, Crystal Structures and Initial Binding Studies (Part II)

**DOI:** 10.1002/open.202300019

**Published:** 2023-07-13

**Authors:** Pierre Seidel, Wilhelm Seichter, Monika Mazik

**Affiliations:** ^1^ Institut für Organische Chemie Technische Universität Bergakademie Freiberg Leipziger Straße 29 09599 Freiberg Germany

**Keywords:** artificial receptors, carbohydrate complexes, fluorescence chemosensors, metal ions, supramolecular synthons

## Abstract

New representatives of 2,4,7‐trisubstituted 9,9‐dialkyl‐9*H*‐fluorenes were prepared and used for crystallographic investigations as well as initial binding studies towards metal ions and carbohydrates. The binding studies, which included ^1^H NMR spectroscopic titrations and fluorescence measurements, demonstrated the ability of the tested fluorene‐based compounds to act as complexing agents for ionic and neutral substrates. Depending on the nature of the subunits of the fluorene derivatives, “turn on” or “turn off” fluorescent chemosensors can be developed. Compounds composed of 4,6‐dimethylpyridin‐2‐yl‐aminomethyl moieties have the potential to be used as sensitive “turn‐on” chemosensors for some metal ions.

## Graphical abstract

New representatives of 2,4,7‐trisubstituted 9,9‐dialkyl‐9*H*‐fluorenes have been prepared and fully characterized, including crystallographic investigations. Initial binding studies towards metal ions and carbohydrates have been carried out and illustrate the prospects of these compounds for sensing purposes and for molecular recognition.

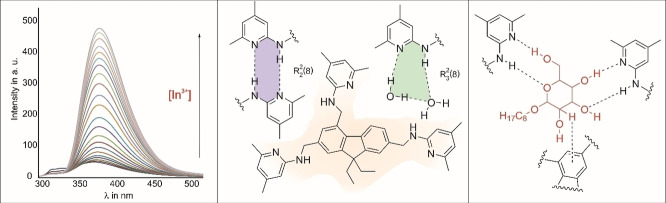

## Introduction

Compounds containing a fluorene moiety have proven to be useful for a wide range of applications, as summarized in many review articles.[^1a^, ^1b^, ^1c^, ^1d^] Among these, fluorene‐based molecules have been developed, that are able to act as artificial receptors for various neutral and ionic substrates. For example, the selective detection of amoxicillin^[2a]^ as well as some di‐ and trivalent metal ions[^2b^, ^2c^, ^2d^] was reported. The fluorescence properties of these fluorene derivatives play an important role in this regard.[^2a^, ^2b^, ^2c^, ^2d^]

Due to the numerous possible applications of fluorenes, the synthesis of new representatives of this class of compounds is the subject of intensive research. Recently, we have reported the syntheses of a series of 2,4,7‐trisubstituted 9,9‐diethyl‐9*H*‐fluorenes^[3]^ as well as of 9,9‐diethyl‐9*H*‐fluorenes bearing four to seven functionalized side‐arms.^[4]^

**Figure 1:**
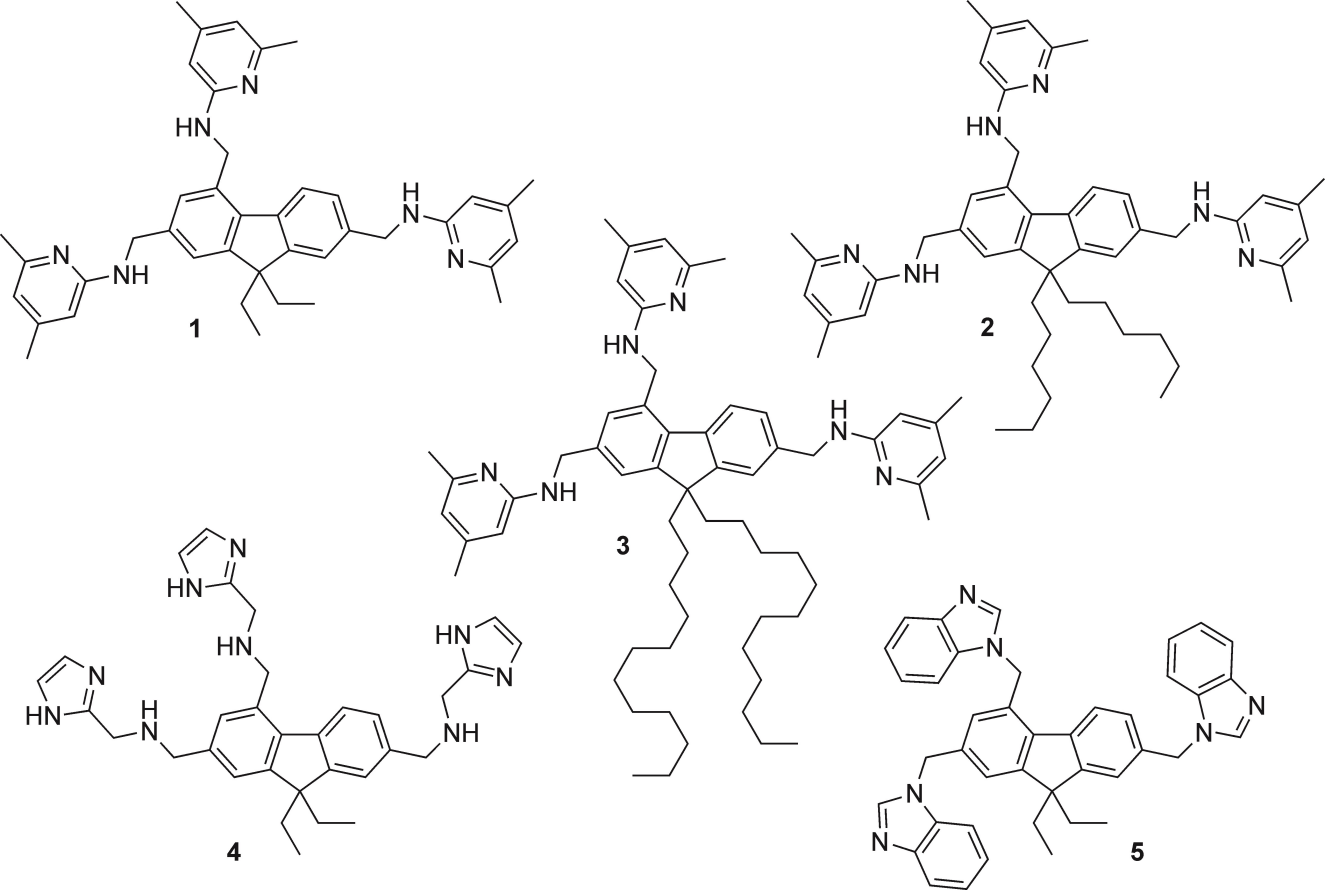
Structures of compounds **1**–**5** considered in this study (the syntheses of compounds **4** and **5** are described in Reference [3]).

Here we describe the synthesis of further representatives of 2,4,7‐trisubstituted 9,9‐dialkyl‐9*H*‐fluorenes containing pyridinyl groups (compounds **1**–**3**, Figure 1). In addition, compounds **4** and **5**, the syntheses of which we have described previously,^[3]^ are also considered in this study.

**Figure 2:**
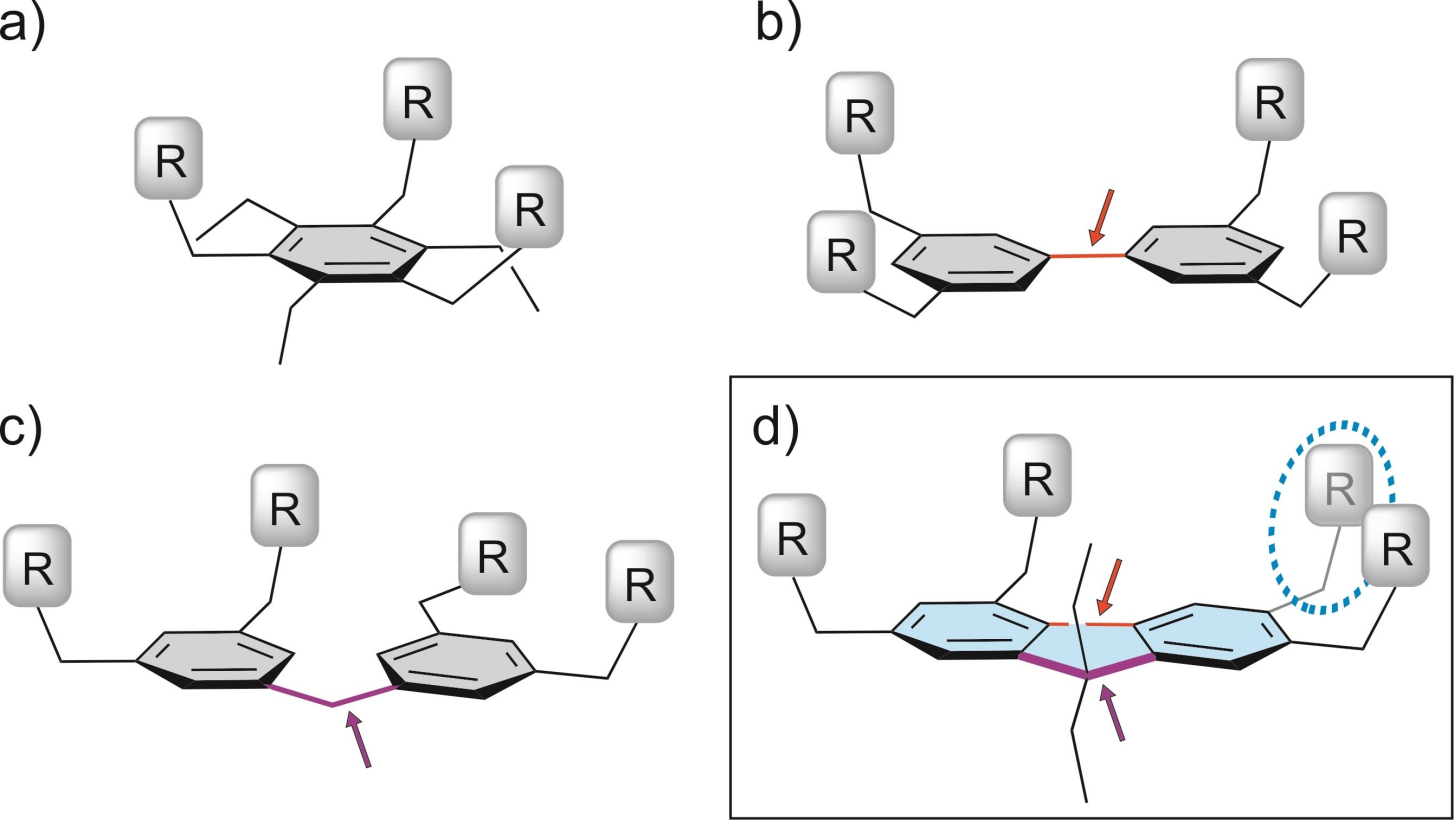
Schematic representation of (a) triethylbenzene‐,[^5^, ^6^] (b) biphenyl‐,^[7a]^ (c) diphenylmethane‐[^7b^, ^7c^] and (d) fluorene‐based receptors with three to four functionalized side‐arms.

These compounds have the potential to act as artificial receptors in analogy to the known receptors with a triethylbenzene‐,[^5a^, ^5b^, ^5c^, ^5d^, ^5e^, ^5f^, ^5g^, ^5h^, ^5i^, ^5j^, ^5k^, ^5l^, ^6a^, ^6b^] biphenyl‐^[7a]^ or diphenylmethane‐based[^7b^, ^7c^] scaffold (Figure 2a–c). It is worth noting that the fluorene backbone (Figure 2d) combines the structural elements of both biphenyl and diphenylmethane scaffolds, as shown by markings in Figure 2.

**Figure 3:**
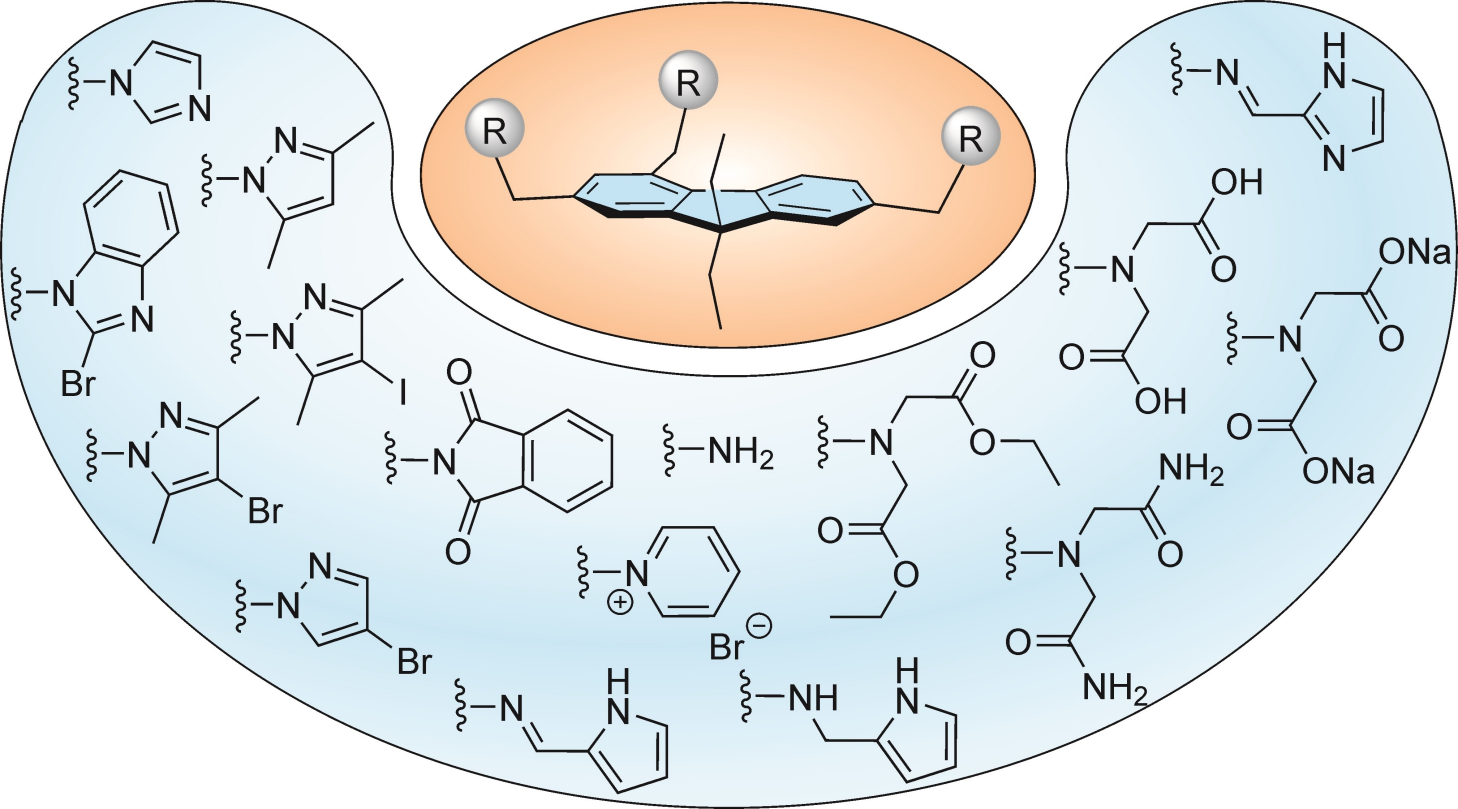
Examples of 2,4,7‐trisubstituted 9,9‐diethyl‐9*H*‐fluorenes, the syntheses of which are reported in Reference [3].

We were interested to investigate how the recognition groups used for the construction of the aforementioned receptors affect the binding properties of fluorene‐based compounds. In conducting the initial binding studies towards carbohydrates and/or metal ions, we considered not only the new compounds with 2‐aminopyridine‐based recognition moieties (compounds **1**–**3**), but also compounds **4** and **5** bearing imidazolyl and benzimidazolyl units (see Figure 1).^[3]^ Examples of further 2,4,7‐trisubstituted 9,9‐diethyl‐9*H*‐fluorenes reported in Reference [3] (Part I) are given in Figure 3.

In compounds **1**–**4**, the heterocyclic groups are linked to the fluorene ring via −CH_2_NH− or −CH_2_NHCH_2_− units, while in **5** they are attached only via a −CH_2_− linker. Depending on the nature of these subunits, “turn on” or “turn off” fluorene‐based fluorescent sensors can be developed. “Turn‐on” sensors are described as desirable in the literature,^[8]^ but their development for metal ions is often challenging. The dialkylation in the 9‐position enhances the thermo‐ and photostability by preventing the oxidation to fluorenone and is necessary to achieve higher quantum yields.^[9]^ Furthermore, this position is also suitable for introducing further recognition units.

In addition, this paper describes the crystal structures of compound **1** and two other 9,9‐diethylfluorene derivatives with *tert*‐butyloxycarbonyl (Boc) protecting groups. The description includes the detailed analysis of the supramolecular interactions in the crystalline state.

## Results and Discussion

### Synthesis of 9,9‐diethylfluorene derivative 1

**Scheme 1:**
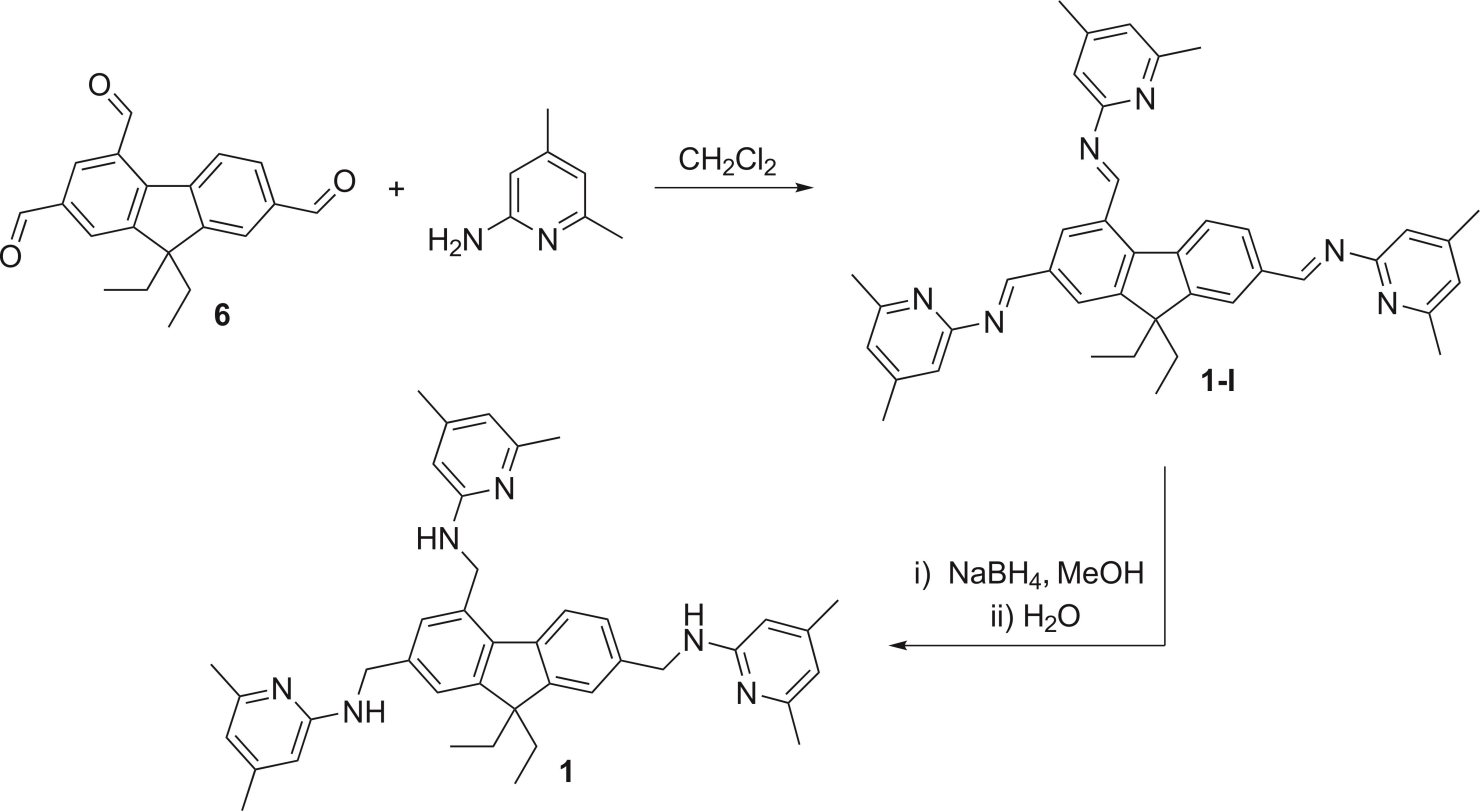
Synthesis of compound **1** using 9,9‐diethyl‐9*H*‐fluorene‐2,4,7‐tricarbaldehyde (**6**) as starting material. Yield over two steps: 36 % of **1**.

**Scheme 2:**
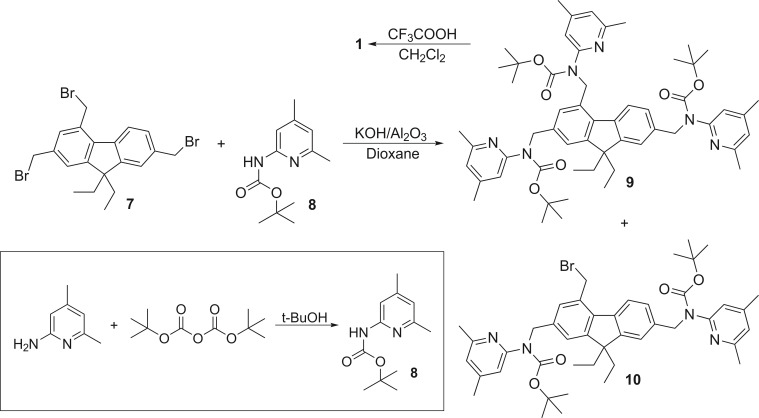
Synthesis of compound **1** using 2,4,7‐tris(bromomethyl)‐9,9‐diethyl‐9*H*‐fluorene (**7**) as starting material. Conditions and yields for **9** and **10**: 16 h, reflux, 39 % of **9**, traces of **10**; 24 h, room temperature, 19 % of **9**, 21 % of **10**. Conditions and yields for the deprotection of **9**: room temperature, 98 % of **1**.

9,9‐Diethyl‐9*H*‐fluorene‐2,4,7‐tricarbaldehyde[^9^, ^10a^, ^10b^] and 2,4,7‐tris(bromomethyl)‐9,9‐diethyl‐9*H*‐fluorene[^9^, ^10a^] were found to be useful starting materials for the synthesis of a wide range of 2,4,7‐trisubstituted 9,9‐diethyl‐9*H*‐fluorenes.[^3^, ^4^] Compound **1** was prepared on the basis of two reaction pathways using the aforementioned starting materials (compounds **6** and **7** in Schemes 1 and 2, respectively).

The first route involves the reaction of 9,9‐diethyl‐9*H*‐fluorene‐2,4,7‐tricarbaldehyde (**6**) with 2‐amino‐4,6‐dimethylpyridine in dichloromethane under reflux conditions to give the corresponding imine‐functionalized fluorene derivative **1‐I**, which was reduced in situ (after addition of methanol) with sodium borohydride (see Scheme 1). Isolation of the product by column chromatography was uncomplicated, since incompletely substituted intermediates are reduced to the corresponding alcohols, which elute significantly worse than the target compound **1**. The desired product was obtained in 36 % yield; the side products could not be isolated in pure form by this route.

As a second synthetic route, the reaction of 2,4,7‐tris(bromomethyl)‐9,9‐diethyl‐9*H*‐fluorene (**7**) with 2‐amino‐4,6‐dimethylpyridine in THF/acetonitrile in the presence of K_2_CO_3_ or Cs_2_CO_3_ was first tested, but did not lead to the target product. This is mainly reasoned by the steric demand of the so‐called bay region.^[11]^ In other words, the functionalization of the 2‐ and 7‐positions of the fluorene core is straightforward, whereas the substitution reaction in the 4‐position of **7** represents a much more challenging task in organic synthesis.

Depending on the nucleophilicity of the reactant used, more drastic reaction conditions, such as higher temperatures, may be required for the efficient proceeding of the complete substitution in the 4‐position of compound **7**, but these also lead to the formation of various side products.

To avoid potential side reactions forming *N,N‐*dialkylated side products, the *N*‐protection of 2‐amino‐4,6‐dimethylpyridine was necessary. The attachment of a *tert*‐butyloxycarbonyl group (Boc protecting group),^[12]^ as shown in Scheme 2, emerged as a reasonable procedure. After deprotonation of the NHBoc group of 2‐(*tert*‐butyloxycarbonylamino)pyridine (**8**) in the presence of KOH, the reaction of the formed nucleophilic species with **7** gave the desired fluorene derivative **9** (Scheme 2).

The latter reaction was performed in dry 1,4‐dioxane at room temperature or under reflux conditions. The removal of the resulting water was carried out by basic alumina (Brockmann I), to which also a catalytic effect is attributed.^[12]^ By varying the reaction parameters, it was possible to optimize the synthesis for **9** and also to find a method to isolate the incompletely substituted derivative **10** by chromatographic workup. The best yield for **9** (39 %) was achieved under reflux conditions, whereas the synthesis at room temperature gave only 19 % of **9**. However, this method proved to be suitable for obtaining the disubstituted derivative **10** with a yield of 21 %. Compound **10** represents a valuable educt for the preparation of fluorene derivatives with differently functionalized side‐arms. It should also be noted that, with appropriate reaction control, the singly substituted derivatives could also be isolated as a mixture of isomers.

The deprotection of **9** to **1** was carried out by using CF_3_COOH in dichloromethane at room temperature and proceeded smoothly with excellent yield (98 %).

### Synthesis of 9,9‐dihexyl and 9,9‐didodecyl fluorene derivatives 2 and 3

**Scheme 3:**
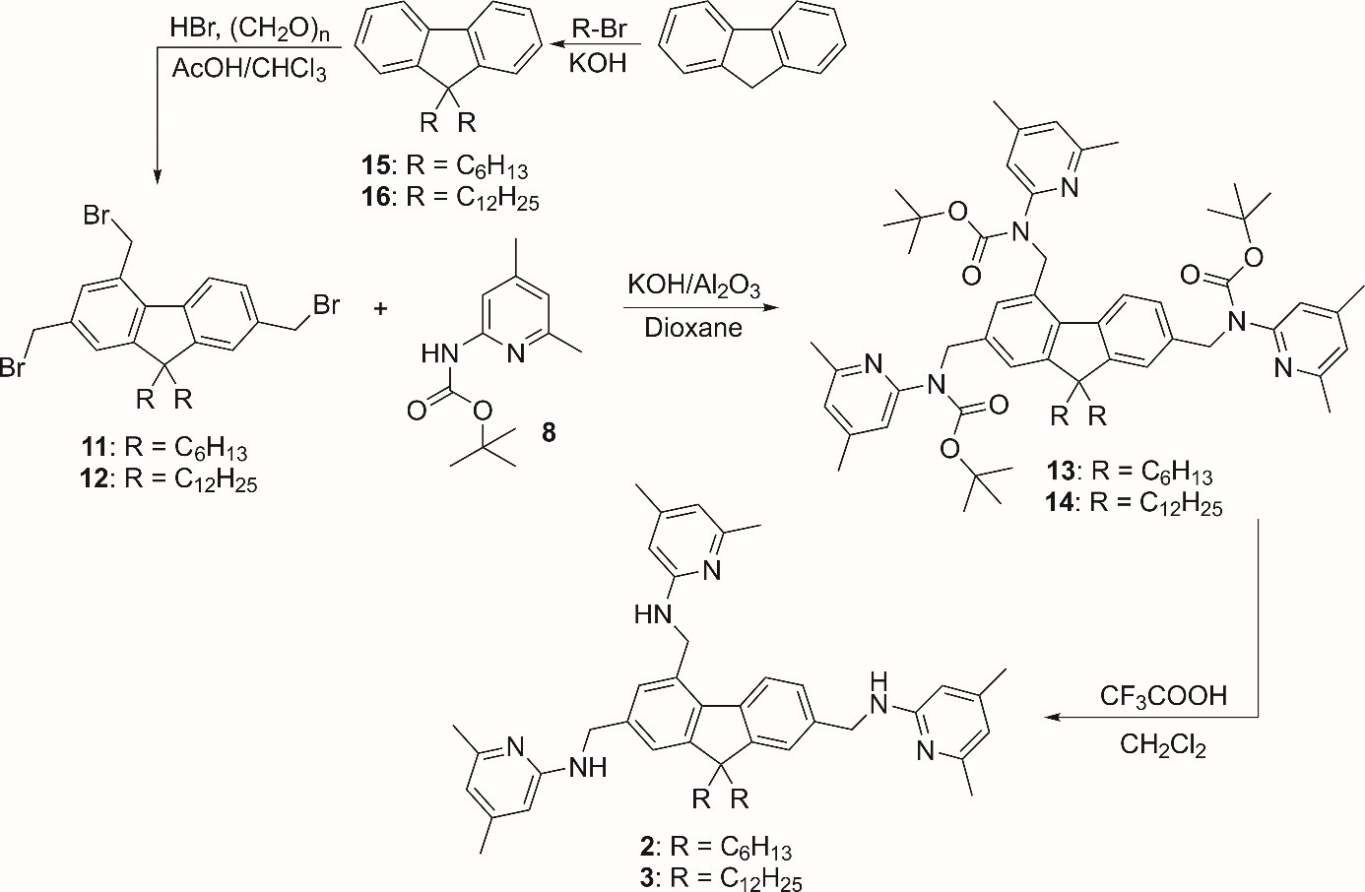
Synthesis of compounds **2** and **3**, using 2,4,7‐tris(bromomethyl)‐9,9‐dihexylfluorene (**11**) and 2,4,7‐tris(bromomethyl)‐9,9‐didodecylfluorene (**12**), respectively, as starting materials. Conditions and yields: 24 h, reflux, 35 % of **13** (starting from **11**) and 40 % of **14** (starting from **12**); 24 h, room temperature, 86 % of **2** (starting from **13**) and 93 % of **3** (starting from **14**).

The two‐step synthetic pathway described for the preparation of compound **1**, using 2,4,7‐tris(bromomethyl)‐9,9‐dialkyl‐9*H*‐fluorene as reactant, was also successfully applied to the synthesis of the derivatives **2** and **3** (Scheme 3). Starting with compounds **11** and **12**, the *tert*‐butoxycarbonyl‐protected 9,9‐dihexyl‐9*H*‐fluorene **13** and 9,9‐didodecyl‐9*H*‐fluorene **14**, respectively, were prepared (with 35 % and 40 % yield). Their deprotection provided the target products **2** (86 %) and **3** (93 %).

The synthesis of **11** and **12** was based on the knowledge gained from the optimization of **7** ^[10a]^ and is also shown in Scheme 3 (for the synthesis of **11**, see also Reference [2b]). The bromomethylation reaction of the 9,9‐dialkylfluorenes **15** and **16** was carried out with the addition of chloroform, which prevented the precipitation of the incompletely reacted intermediates.

It should be noted that the length of the alkyl chains affects not only the solubility of the compounds but also the reactivity of the aromatic system. Reference should be made to the intramolecular CH⋅⋅⋅π interactions that occur between the β‐CH_2_ groups and the fluorene core, as has been observed in crystal structures.^[13]^ Increasing the length of the alkyl chains most likely leads to steric hindrance at the fluorene and consequently to a reduction in the reaction rate in adjacent positions.

### Crystal structures of compounds 1, 9 and 10

**Figure 4:**
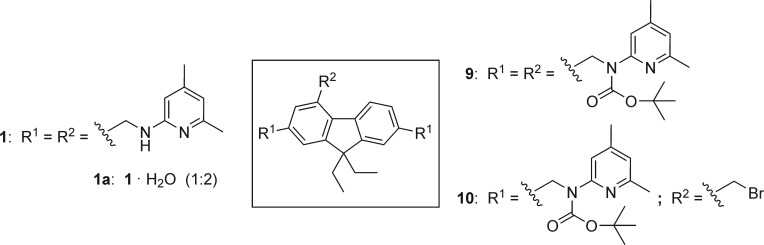
Structures of compounds **1**, **9** and **10**, the crystal structures of which are described in this paper (**1 a** represents the hydrate of **1**).

In the course of our experimental work, we succeeded in growing crystals of three 2,4,7‐trisubstituted derivatives of 9,9‐diethyl‐9*H*‐fluorene. Schematic representations of the molecular structures of compounds **1**, **9** and **10** are shown in Figure 4. Crystals suitable for X‐ray structural analysis were obtained by slow evaporation of the solvent from a solution of the respective compound. The crystallographic data, geometric molecular parameters and information regarding intermolecular interactions present in the crystals are summarized in Tables S1–3 (the numbering of the ring positions of the fluorene unit does not refer to the IUPAC nomenclature). To simplify the structural descriptions, the aromatic building blocks of the molecules were marked by large letters in the figures showing the molecular structures.

**Figure 5:**
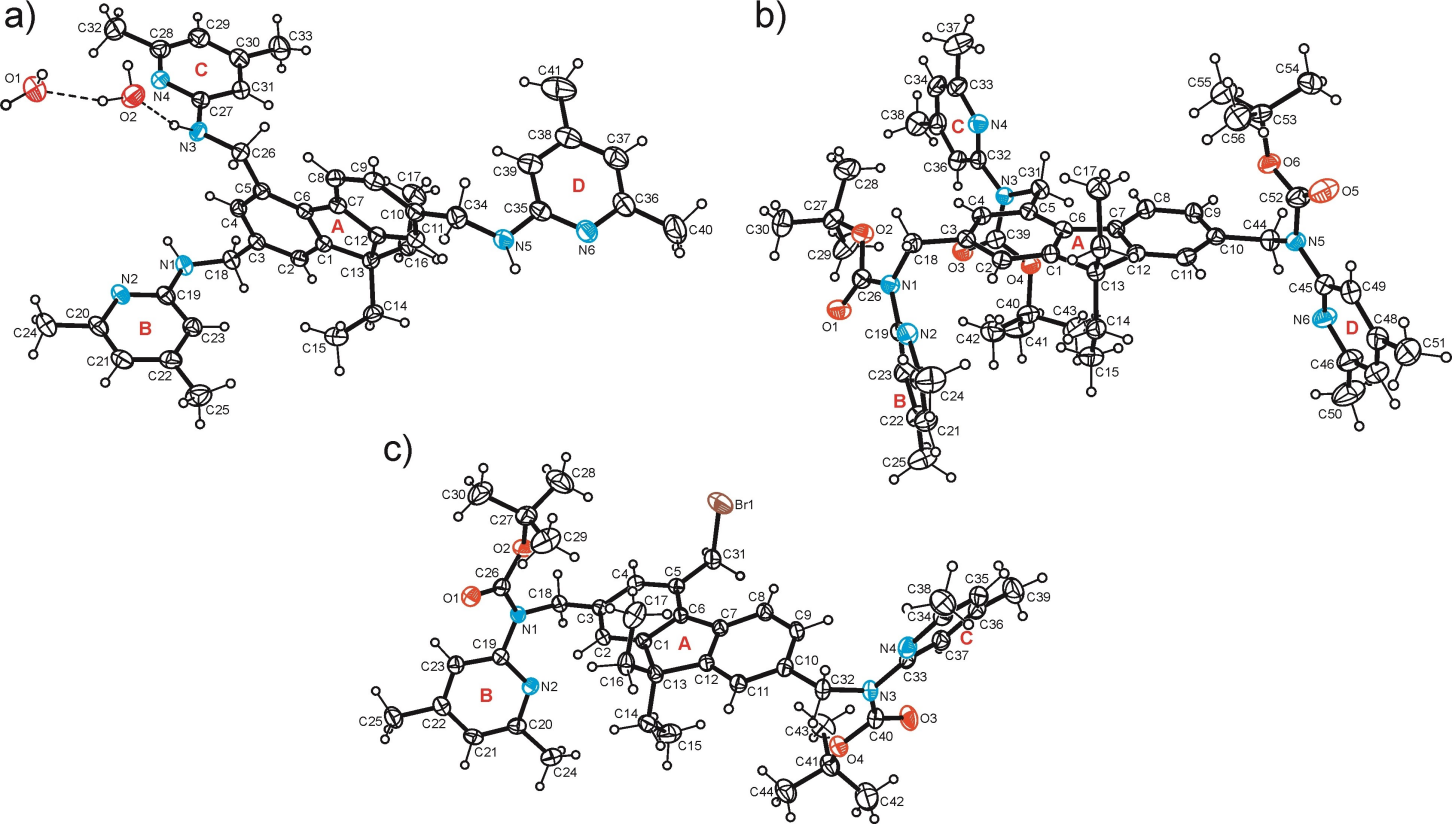
Perspective views (ORTEP diagrams) of the molecular structures of **1** ⋅ H_2_O (1 : 2; **1 a**), **9** (b) and **10** (c) including atom numbering and ring specification. Displacement ellipsoids are drawn at the 50 % probability level. The numbering of the ring positions of the fluorene unit does not refer to the IUPAC nomenclature.

**Figure 6:**
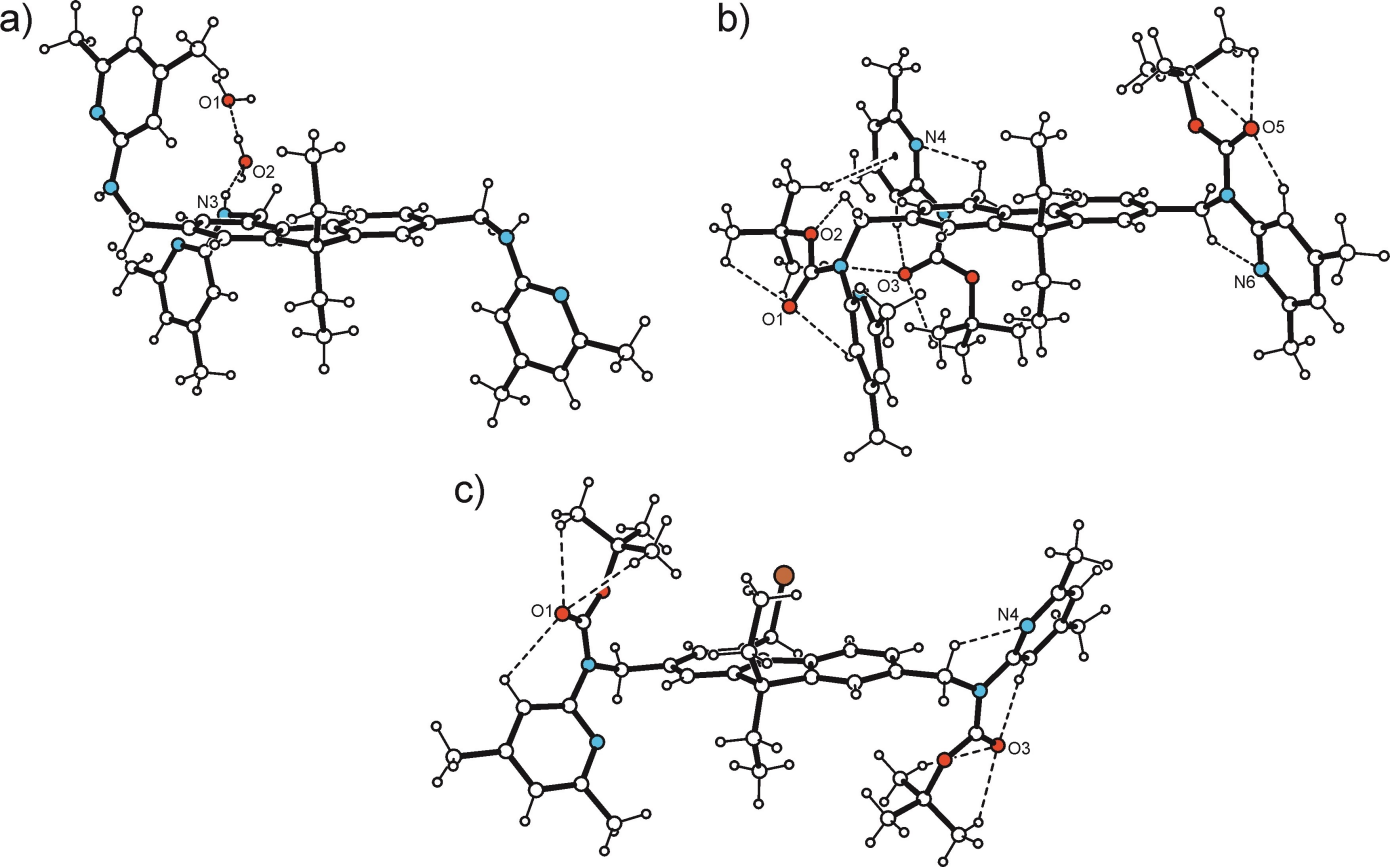
Ball‐and‐stick representations (side views) of the molecular structures of **1** ⋅ H_2_O (1 : 2; **1 a**), **9** (b) and **10** (c) including atom numbering of coordinating atoms. Broken lines represent hydrogen bonds.

**Figure 7:**
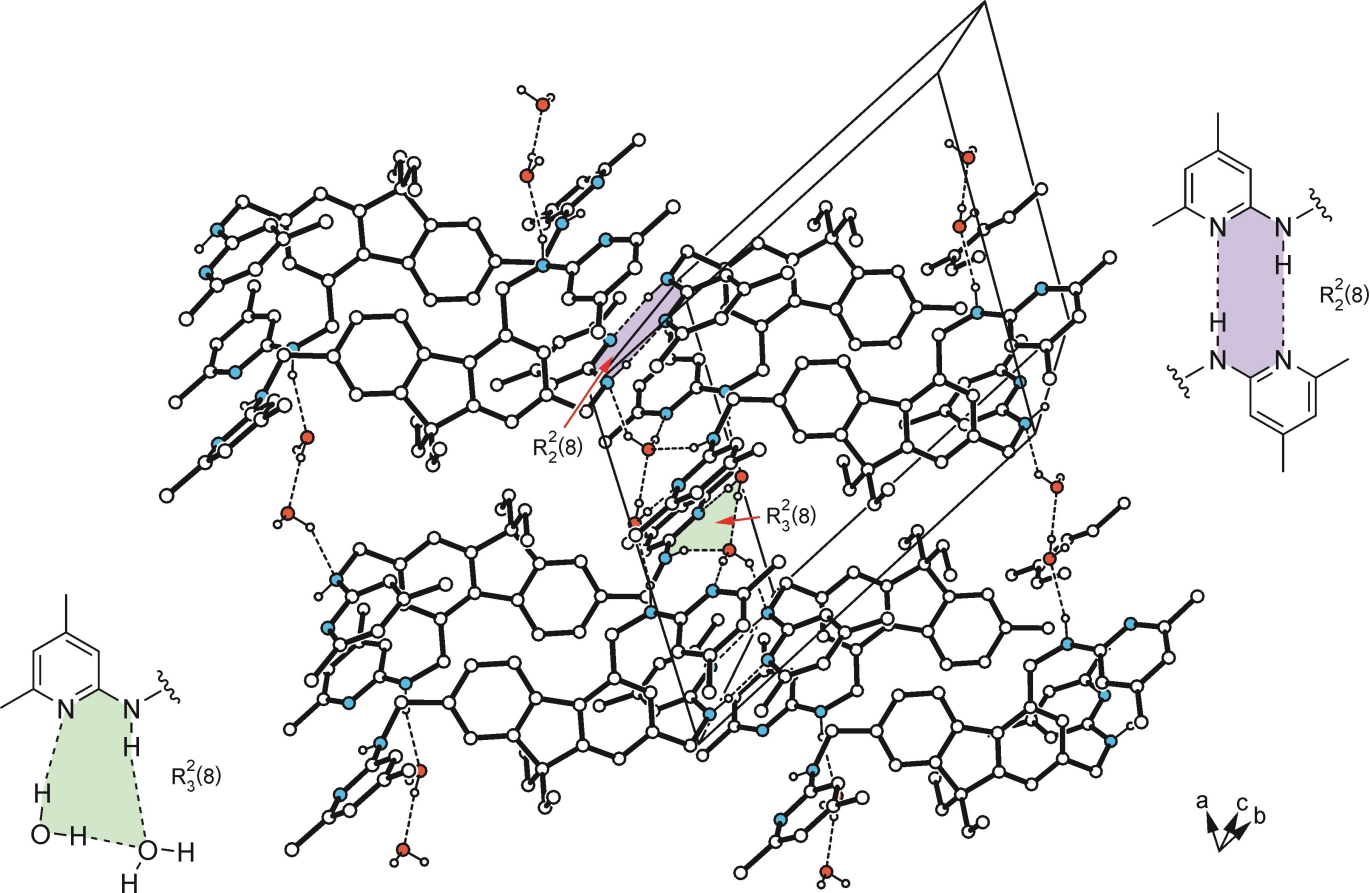
Packing diagram of **1** ⋅ H_2_O (1 : 2; **1 a**). Broken lines represent hydrogen bond interactions. The cyclic supramolecular synthons are marked by color highlighting.

The colorless crystals of compound **1** obtained from acetonitrile were found to be a dihydrate of the space group *P*‐1 (*Z*=2), with the asymmetric unit of the cell containing the 1 : 2 host‐guest complex of the structure shown in Figure 5a. The three heterocyclic units of the molecule adopt a “*two‐up*/*one‐down”* orientation with respect to the plane of the fluorene framework, with the two 4,6‐dimethylpyridin‐2‐yl‐aminomethyl groups linked to the C(3) and C(5) atoms located on opposite sides of the fluorene (see Figure 6a). The three pyridine rings are inclined at angles of 78.9(1), 75.4(1), and 83.2(1)° to the least‐squares plane of the fluorene unit. These values correspond to torsion angles of −64.4(2), −80.7(2), and 89.1(2)° for the atomic sequences C_fluorene_−C−N−C_pyridine_. Due to packing forces and coordination effects, the fluorene unit exhibits a noticeable bending, with its aromatic rings forming an angle of 8.8(1)°. The linkage of the complexes in the crystal is mediated primarily by the water molecules giving rise to formation of a supramolecular ring motif of the structure R_3_^2^(8)^[14]^ that comprises O−H⋅⋅⋅O [*d*(H⋅⋅⋅O) 1.96(1) Å], N−H⋅⋅⋅O [*d*(H⋅⋅⋅O) 2.06(1) Å] and O−H⋅⋅⋅N interactions [*d*(H⋅⋅⋅N) 1.97(1) Å] (see Figure 7). The connection between host molecules occurs through N−H⋅⋅⋅N_pyridine_ interactions (Figure 7), thus forming an inversion‐symmetric synthon of the graph set R_2_^2^(8). The pattern of intermolecular interactions is completed by numerous C−H⋅⋅⋅π contacts^[15]^ [*d*(H⋅⋅⋅*Cg*) 2.72–2.89 Å] with all aromatic building blocks acting as receptors.

Compound **9** represents the Boc‐protected analogue of **1** and crystallizes, like **1**, in the space group *P*‐1 with two molecules in the unit cell [Figure 5b; crystal growth occurred from *n*‐hexane/CH_2_Cl_2_ (10 : 1)]. The molecular conformation in the present crystal structure is stabilized by a large number of intramolecular H‐bonds (Figure 6b). The carbonyl oxygen atom of the respective Boc group acts as a trifurcated acceptor for the formation of intramolecular C−H⋅⋅⋅O^[16]^ bonds [*d*(H⋅⋅⋅O) 2.13–2.46 Å]. In addition, the N(2), N(4), N(6) and O(2) atoms are involved in the formation of intramolecular C−H⋅⋅⋅N [*d*(H⋅⋅⋅N) 2.16, 2.32, 2.35 Å] and C−H⋅⋅⋅O bonds [*d*(H⋅⋅⋅O) 2.22 Å], respectively. The twist angles of the heterocyclic units with respect to the fluorene plane are 85.8(1), 86.3(1) and 76.8(1)°. While the Boc‐N‐Pyr groups attached to C(5) and C(10) adopt an approximately antiparallel arrangement and their Boc units are located on opposite sides of the fluorene framework, the unit linked to C(3) is rotated out of the fluorene plane to only a small extent.

The steric influence of the *tert*‐butyl groups and the accompanying high degree of intramolecular interactions involving the strong acceptor sites of the molecule are likely the reason for the weak cross‐linking of the molecules in the crystal. Intermolecular interactions are therefore limited to C−H⋅⋅⋅π [*d*(H⋅⋅⋅*Cg*) 2.59 Å] and offset π⋅⋅⋅π^[17]^ (face‐to‐face) interactions [*d*(*Cg*⋅⋅⋅*Cg*) 3.922(3) Å, slippage 1.322 Å] between the pyridine rings B and D as well as weak C−H⋅⋅⋅O bonds [*d*(H⋅⋅⋅O) 2.57 Å] involving the atom O(5). The packing diagram of the crystal structure is shown in Figure S1 in the Supporting Information.

**Figure 8:**
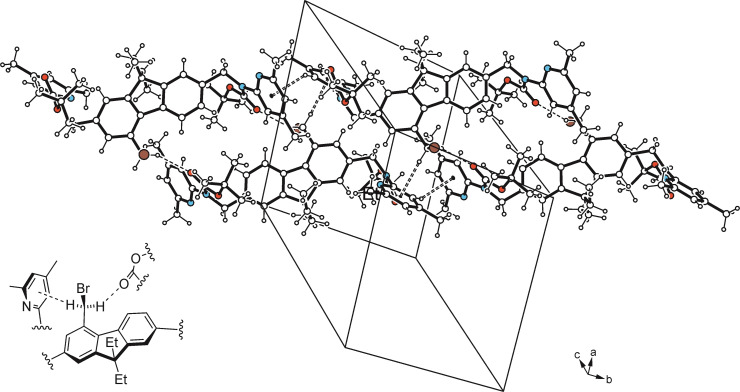
Packing diagram of **10**. Broken lines represent hydrogen bonds, broken double lines represent C−H⋅⋅⋅π interactions.

Crystal growth of **10** from *n*‐hexane/CH_2_Cl_2_ (10 : 1) yields colorless platelets of the space group *P*‐1 with one molecule in the asymmetric unit of the cell. In the crystal, the two Boc‐protected aminopyridine units adopt nearly opposite orientations, so that their *tert*‐butoxycarbonyl moieties are located on different sides of the fluorene unit (Figures 5c, 6c). The pyridine rings are inclined at angles of 80.6(1) and 75.3(1)° with reference to the plane of the fluorene group. The two Boc‐protected aminoaryl units are twisted to a different degree, as expressed by the torsion angles of −140.3(2) and −154.2(2)° for the two atomic sequences C−N_amine_−C−N_pyridine_ which can be regarded as an explanation for the fact that only the N atom of the pyridine ring designated C is involved in an intramolecular C−H⋅⋅⋅N bond. In addition, the carbonyl oxygen atoms O(1) and O(3) act as trifurcated binding sites for intramolecular C−H⋅⋅⋅O bonds involving H atoms of the *tert*‐butyl groups and an aryl H atom. The crystal of **10** consists of one‐dimensional aggregates running parallel to the crystallographic [111] plane, in which the molecules are only weakly cross‐linked (Figure 8).

Some views of the superpositions of molecules **1**&**9** and **9**&**10** are given in Figure S2 in the Supporting Information.

### Initial binding studies towards metal ions and carbohydrates: Fluorescence experiments and ^1^H NMR spectroscopic titrations

#### Fluorescence properties of compound 1 in various solvents

Solvents influence both the complexation process itself and the fluorescence emission of fluorophores. The behavior of compound **1** in various organic solvents was examined using dimethyl sulfoxide, chloroform, diethyl ether, dichloromethane, acetonitrile, and methanol. To investigate the binding properties of **1** towards metal ions in water (for details, see the description of the binding studies), the addition of methanol, for example, is necessary due to the solubility behavior of **1**. Therefore, measurements were also performed in methanol/water mixtures.

**Figure 9:**
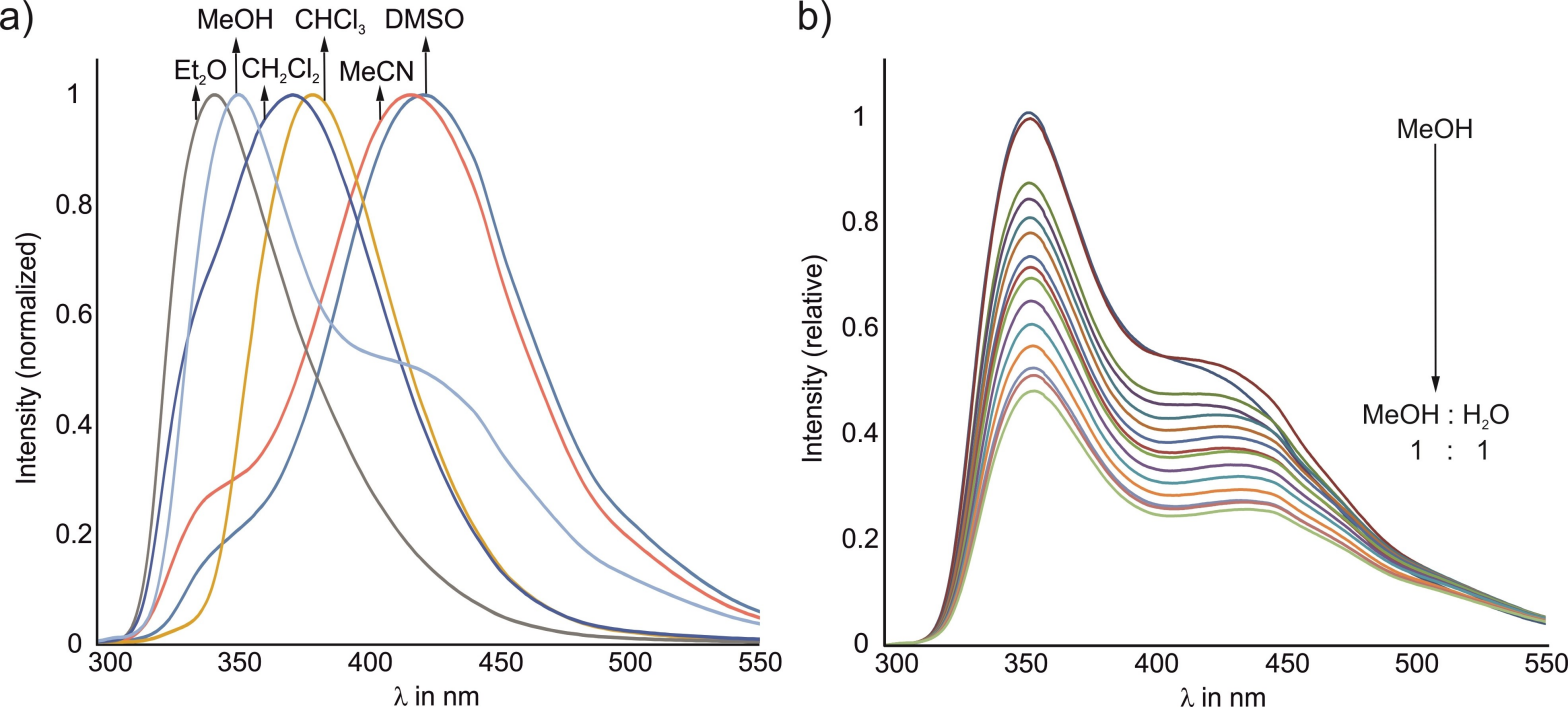
Fluorescence spectra of **1** (a) recorded in various organic solvents (normalized to maximum intensity) and (b) in methanol/water mixtures (relative intensities).

All emission spectra were recorded at an excitation wavelength of 250 nm for all solvents, except for DMSO, where an excitation wavelength of 260 nm had to be chosen. The concentration of **1** was adjusted to 4 ⋅ 10^−5^ mol/L for all samples. As expected, the experiments showed strong solvent effects on the fluorescence emission of compound **1**, which are illustrated in Figure 9a. The different number of observable bands also indicates solvent‐dependent aggregation processes (at the concentration mentioned above).

The results of the measurements in methanol/water mixtures demonstrate that apart from the drop in fluorescence intensity, there is no fundamental change in the spectrum in methanol as a function of the water content (Figure 9b).

**Figure 10:**
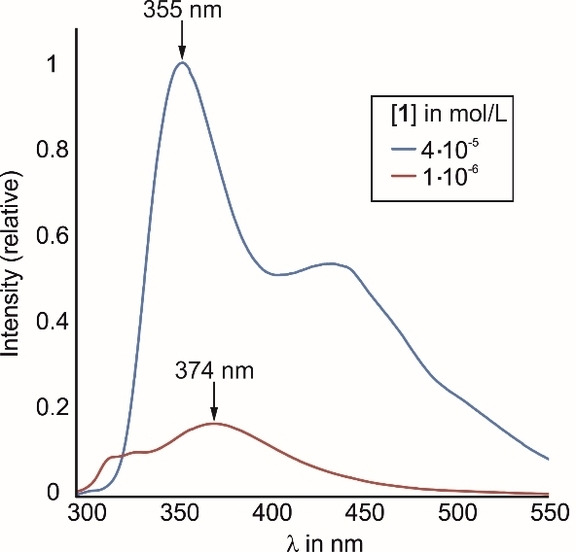
Comparison of the emission spectra (relative intensities) of differently concentrated solutions of **1** (c=4 ⋅ 10^−5^ or 1 ⋅ 10^−6^ mol/L).

A frequently observed phenomenon in spectra of concentrated fluorophore solutions is the formation of excimers, which is reflected in a band that is red‐shifted with respect to the monomer emission. Since fluorene is particularly prone to self‐aggregation, a diluted sample was used to investigate whether this also occurs in the present case. Figure 10 clearly shows that reducing the concentration from 4 ⋅ 10^−5^ to 1 ⋅ 10^−6^ mol/L leads to the disappearance of the band at 436 nm, which can thus be identified as an excimer band. Furthermore, the dimethylpyridine monomer band is red‐shifted from 355 nm to 374 nm, indicating that the environment around the fluorophores becomes more polar as a result of dilution.

#### Binding studies of compounds 1, 4 and 5 with selected cations

The choice of ionic substrates is based on the ecological and economic relevance of the elements and includes the chlorides, nitrates and sulfates of Mg^2+^, Ca^2+^, Fe^2+^, Co^2+^, Cu^2+^, Ni^2+^, Sn^2+^, Al^3+^, Fe^3+^, In^3+^, V^3+^, Eu^3+^, Mo^5+^. Furthermore, the influence of H^+^, Na^+^, K^+^ on the complexing properties of the tested compounds was examined. The discrepancy between the solubility of the corresponding salts and that of the fluorene derivatives **1**, **4** and **5**, bearing three heterocyclic groups, required extensive experiments to find the appropriate water‐containing medium.

The good solubility of the selected 9,9‐diethylfluorenes **1**, **4** and **5** in methanol allowed mixing with water up to a volume ratio of 1 : 1 methanol/water below a concentration of 10^−4^ mol/L, without causing precipitation. A suitable excitation wavelength was determined using UV/Vis spectroscopy, with all samples providing reproducibly good emission spectra at 250 nm.

**Figure 11:**
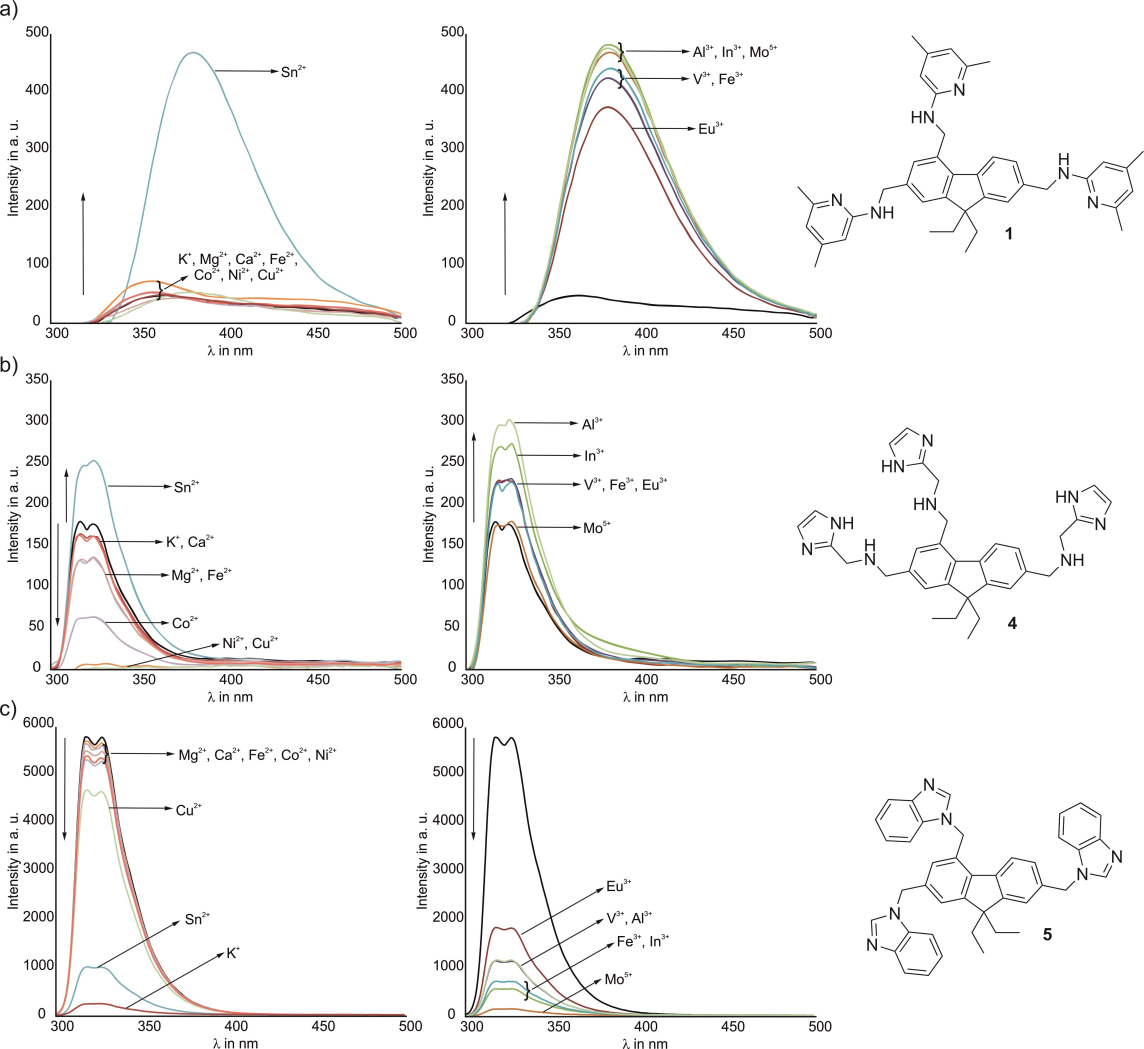
Change in fluorescence spectra of the fluorene derivatives **1**, **4**, and **5** upon addition of different cations (1 equiv.) in methanol/water (1 : 1, v/v).

First insights into the fluorescence and complexation behavior of compounds **1**, **4** and **5** were provided by screening with the mentioned salts by using a microtiter plate. In each case, the concentration of the corresponding fluorene derivative and the metal ion were set to 5 ⋅ 10^−5^ mol/L. This was necessary to achieve an acceptable signal‐to‐noise ratio in the measurements with the well plate reader. This resulted in the first interesting findings (Figure 11; UV/Vis spectra are given in Figure S3 in the Supporting Information).

First of all, the extraordinarily high fluorescence intensity of **5** compared to the other two compounds should be emphasized, which is influenced by several factors. On the one hand, compound **5** does not have NH functions that can be responsible for quenching the fluorescence of the fluorophores via a PET mechanism,^[18a]^ as it is the case with **1** and **4**. Furthermore, the lower flexibility of the functionalized side‐arms should be mentioned.

In the case of compound **5**, the affinity for K^+^ is particularly impressive, but also for higher‐valent ions such as Mo^5+^. Compound **1** possesses pyridinyl groups, which are themselves fluorophoric. This compound showed a completely different emission behavior compared to **4** and **5**. In the presence of the higher‐valent ions, the fluorescence of **1** is enhanced many times over. In the case of compound **4**, the complexation of Ni^2+^ and Cu^2+^ is again indicated by a complete quenching of the fluorescence. The quenching probably results from the aforementioned PET from the respective fluorophore to the metal ion.

Due to its fluorescence behavior, compound **1** has the potential for the development of “turn‐on” sensors,^[18b]^ which often enable a significantly higher sensitivity than corresponding “turn‐off”^[18c]^ variants and thus represent attractive candidates for analytical purposes.

#### Interactions of 1 and 3 with InCl_3_

Since the screening revealed a clear affinity of **1** for trivalent metal ions, InCl_3_ was chosen as a substrate for detailed analysis using fluorescence titrations.

Indium belongs to strategic elements and the development of complexing agents for indium ions, e. g., for use in solvent extractions,[^19a^, ^19b^, ^19c^, ^19d^, ^19e^, ^19f^, ^19g^] is of great importance. Furthermore, the detection of In^3+^ is also important^[20]^ because of its toxicity.[^20^, ^21a^, ^21b^, ^21c^] For example, indium ions have been reported to cause severe lung damage and may promote the development of fibrosis.^[21c]^

**Figure 12:**
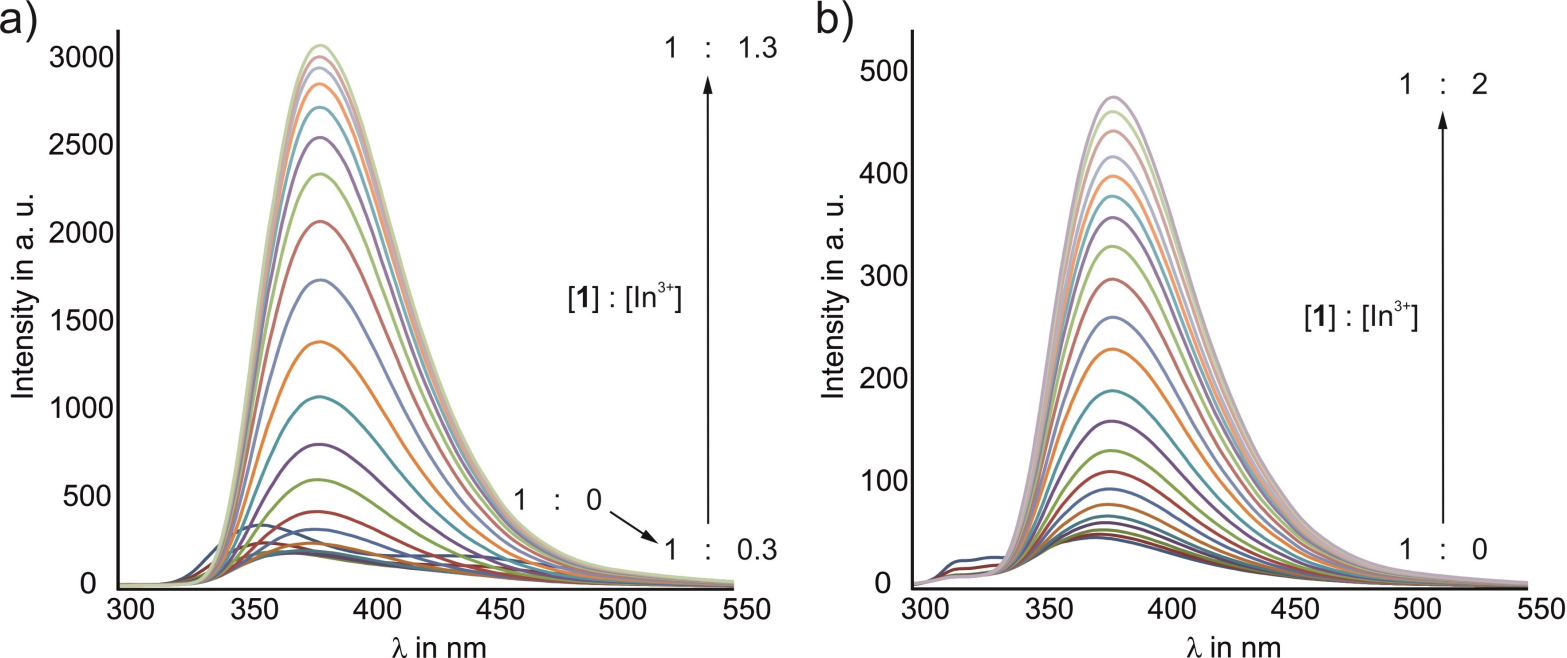
Results of fluorescence titrations of **1** with InCl_3_ at a receptor concentration of 4 ⋅ 10^−5^ mol/L (a) and 1 ⋅ 10^−6^ mol/L (b); see also Figure S4 in Supporting Information.

The titration experiments of compound **1** with InCl_3_ were performed using a flow cell with an associated peristaltic pump, so that a large sample volume (40 mL) was possible. The solution of the fluorene derivative in methanol/water (1 : 1, v/v) was prepared with a concentration of 4 ⋅ 10^−5^ mol/L (3 ⋅ 10^−4^ mol/L HEPES buffer) or 1 ⋅ 10^−6^ mol/L (1 ⋅ 10^−5^ mol/L HEPES buffer), respectively. The appropriately concentrated indium salt solution was added, as given in the Supporting Information (see Tables S4 and S5). The results are shown in Figure 12.

The previously identified fluorescence properties of compound **1**, which differ significantly in concentrated and dilute solution, are also reflected by the results of the titration experiments. At a concentration of the fluorene derivative of 4 ⋅ 10^−5^ mol/L, where self‐aggregation is more pronounced, the fluorescence was initially quenched and a red shift was observed up to an addition of 0.3 equivalents of In^3+^. This indicates a gradual breakdown of the aggregate structure caused by the complexation of the ions. When the concentration of the indium ions was further increased, the intensity enhanced again, resulting in only a single emission band. The saturation point was identified at a receptor‐to‐substrate ratio of 1 : 1.3.

In the case of the diluted sample, the addition of the indium salt directly led to an increase in intensity and a much weaker red shift. Moreover, the saturation range was reached at a receptor‐substrate ratio of 1 : 2.

In both series of experiments, the emission maximum at the last measurement point was between 380–382 nm, indicating that upon complexation of In^3+^, the fluorescent species should be of the same type in concentrated and in dilute solutions.

The measurements provided evidence for the formation of complexes with a complicated stoichiometry, which prevented the accurate determination of the binding constants. However, the detection of In^3+^ in a concentration range of 10^−7^ mol/L by compound **1** indicates a high sensitivity and strong complex formation.

For comparing the properties of **1** and its analogue bearing dodecyl groups in the 9‐position of the fluorene ring, samples were prepared with derivative **3** (see Table S6) and analyzed according to the above‐detailed procedure. In principle, the behavior is similar to that of **1**, but the significantly stronger increase in the intensity of the emission maximum should be emphasized (see Figures S5 and S6 in the Supporting Information).

#### Initial studies on carbohydrate binding by compound 1

In addition to the binding studies with selected cations, the interactions of compound **1** with carbohydrates were investigated. The results of these initial binding experiments with a fluorene derivative contribute to our systematic studies on the molecular recognition of carbohydrates by various artificial receptor molecules. Within the scope of these systematic studies, we are developing acyclic[^5^, ^6^] and macrocyclic[^22a^, ^22b^, ^22c^, ^22d^] receptor molecules that can both mimic the key protein‐carbohydrate interactions^[23]^ and have a relatively simple structure to synthesize. Not only the X‐ray structural analyses of protein‐carbohydrate complexes, but also the crystal structures of complexes of artificial receptors with carbohydrates obtained in the course of our studies^[24]^ served as a source of ideas for the design of new receptor structures.

In the case of the acyclic compounds, effective binding of the carbohydrate substrate is realized by a receptor architecture with a central aromatic moiety bearing three or more functionalized side‐arms as recognition groups (see Figure 2a–c). The binding properties of these types of compounds can be fine‐tuned by varying these structural subunits.

In addition to the functional groups used in nature for molecular recognition of carbohydrates, structural analogs of these natural recognition groups have also been incorporated into the receptor structure, such as the 2‐aminopyridine moiety,^[6]^ which can be considered as a heterocyclic analog of the primary amide group of the asparagine/glutamine side chain.^[25]^

Biphenyl,^[7a]^ diphenylmethane[^7b^, ^7c^] or benzene[^5a^, ^5b^, ^5c^, ^5d^, ^5e^, ^5f^, ^5g^, ^5h^, ^5i^, ^5j^, ^5k^, ^5l^, ^6a^, ^6b^] units were used as the central aromatic platform in the construction of the receptor structures. The aromatic core has both the task of arranging the recognition units in such a way that a three‐dimensional recognition of the carbohydrate substrate is possible, and to enable the formation of CH⋅⋅⋅π interactions with the carbohydrate‐CH units. The larger aromatic moieties are particularly important for the construction of oligosaccharide receptors.

Fluorene‐based carbohydrate receptors of the above type, to our knowledge, have not yet been developed and are part of our current research (Figure 2d). The binding properties of such receptor molecules are influenced by many factors, including the number and type of recognition groups and their position in the fluorene ring. The fluorene derivatives are expected to be able to recognize both mono‐ and oligosaccharides, with a strong preference for the oligosaccharides.

For initial studies on carbohydrate binding by compound **1**, the preference for binding di‐ vs. monosaccharides was planned to be investigated in CDCl_3_. To compare the binding properties of **1** with those of the previously examined receptors, which were found to show di‐ vs. monosaccharide preference, dodecyl β‐d‐maltoside, dodecyl α‐d‐maltoside and octyl β‐d‐glucopyranoside were selected as substrates. However, due to solubility problems, the studies with disaccharides were not feasible. For example, unlike a biphenyl derivative containing four aminopyridine‐based recognition units,^[7a]^ compound **1** is unable to dissolve dodecyl β‐d‐maltoside in CDCl_3_, which is poorly soluble in this solvent. This shows that the interactions between the four‐fold substituted biphenyl‐based receptor^[7a]^ and the tested disaccharide are more favourable under the selected experimental conditions than in the case of compound **1**, bearing only three heterocyclic recognition units.

To perform the binding studies, further structural variations of the fluorene backbone are required, including functionalization of additional positions of the fluorene ring (e. g., the 6‐position) and/or variation of substituents in the 9 position, as shown in Figure S7 in Supporting Information. The use of other carbohydrate substrates (e. g., cellobioside) is an important component of the planned studies in organic and aqueous media.

The interactions of the fluorene derivative **1** with the well‐soluble octyl β‐d‐glucopyranoside (**βGlc**) were investigated by ^1^H NMR spectroscopic titrations (Table S7), which confirmed our expectations regarding the binding affinity of **1** towards the monosaccharide. By using the NMR method, structural information about the formed complexes can be obtained. The complexation‐induced chemical shifts of selected signals of **1** are given in Figure S8. As expected, the changes are not very pronounced, but provided indications for the binding mode of the substrate **βGlc** to **1**, which was also suggested by molecular modelling calculations.

**Figure 13:**
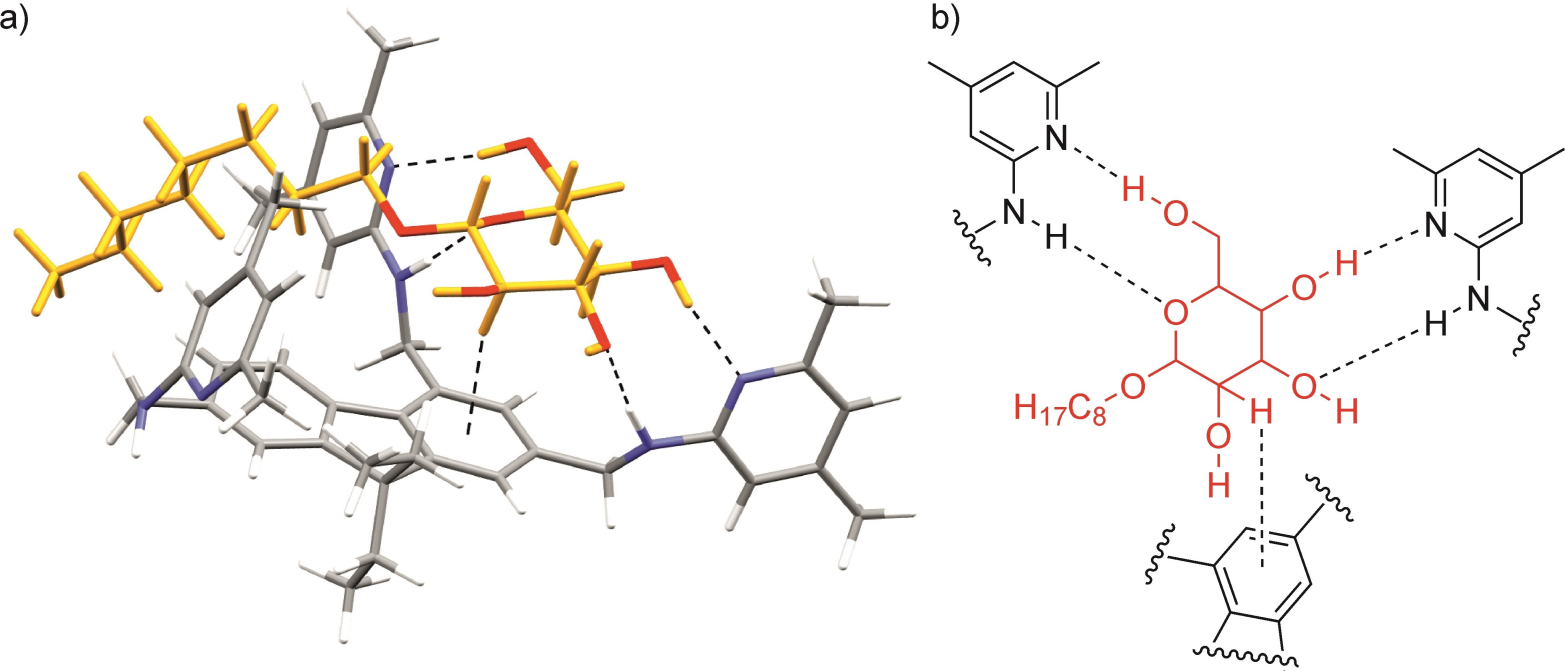
Energy‐minimized structure of the 1 : 1 complex of compound **1** with octyl β‐D‐glucoside (**1** ⋅ **βGlc**) and schematic representation of the non‐covalent interactions that stabilize the complex. MacroModel V.9.8, OPLS_2001 force field, MCMM, 50000 steps; Color code: receptor N, blue; receptor C, grey; sugar O, red; sugar C/H, yellow.

As indicated by molecular modelling calculations and the results of NMR measurements, the monosaccharide is located in the neighborhood of the heterocyclic moieties attached to the 2‐ and 4‐positions of the fluorene ring and is involved in the formation of hydrogen bonds and CH⋅⋅⋅π interactions, as shown in Figure 13. For example, the 3‐, 4‐, and 6‐OH groups and the ring oxygen of the glucopyranoside participate in hydrogen bonds with the two aminopyridine subunits of the receptor; the bidentate hydrogen bonds include 4‐OH⋅⋅⋅N_pyr_/N−H⋅⋅⋅OH‐3 and 6‐OH⋅⋅⋅N_pyr_/N−H⋅⋅⋅O_ring_ interactions. Such hydrogen bonding interactions were also observed by us in the crystal structures of the complexes of 1,3,5‐trisubstituted 2,4,6‐trialkylbenzenes and glucopyranosides, including methyl α‐ and β‐d‐glucopyranoside as well as octyl β‐d‐glucopyranoside.[^24a^, ^24b^, ^24c^]

Analysis of the ^1^H NMR titration data^[26]^ revealed a weak binding constant of 1460 M^−1^ for **1** ⋅ **βGlc**, as expected. Similar binding affinity was exhibited by the previously studied triethylbenzene derivative with two aminopyridine‐based recognition units,^[27a]^ while the analogue with the third heterocyclic recognition moiety^[27b]^ proved to be a much stronger receptor for **βGlc** under similar experimental conditions (see Figure S9, Supporting Information).

In the case of the fluorene‐based compounds, the incorporation of a fourth heterocyclic recognition group into the fluorene backbone (see Figure S10 for an example) is expected to be responsible for the formation of 1 : 2 receptor‐monosaccharide complexes, as observed in the case of the aforementioned biphenyl derivative. Such compounds have multiple donor/acceptor sites for hydrogen bonding and possess a cavity of suitable size and shape for disaccharide binding. Therefore, they are expected to be powerful receptors for disaccharides, such as, for example, cellobioside (see Figure S10).

Fluorene derivatives often have a strongly pronounced tendency to self‐aggregate, which must be considered very carefully when designing the molecules and performing the binding studies. The synthesis of numerous new fluorene‐based compounds, the design of which has been influenced by the above factors, and which should be suitable for binding studies towards carbohydrates in organic and/or aqueous media is currently underway.

## Conclusions

The series of 2,4,7‐trisubstituted 9,9‐diethyl‐9*H*‐fluorenes bearing different functional groups, the syntheses of which we have recently described,[^3^, ^4^] is extended by the 9,9‐dialkylfluorenes synthesized in the present studies (target molecules **1**–**3** and intermediates **9**, **10**, **13** and **14**). The new fluorenes, which contain aminopyridine‐based recognition units and have ethyl, hexyl or dodecyl groups in the 9‐position of the fluorene backbone, were prepared using 2,4,7‐tris(bromomethyl)‐9,9‐dialkyl‐9*H*‐fluorenes and 9,9‐diethyl‐9*H*‐fluorene‐2,4,7‐tricarbaldehyde as educts.

In addition to the syntheses, the manuscript presents the molecular structures of three fluorene derivatives in the crystalline state (compounds **1**, **9** and **10**) and describes the supramolecular motifs observed in the crystal structures in detail.

The initial binding studies towards metal ions and carbohydrates, carried out with compounds **1**, **3** and the previously prepared derivatives **4** and **5**, demonstrate the potential of 9,9‐dialkylfluorenes bearing appropriately functionalized side arms to act as receptor/chemosensor molecules for various ionic and neutral substrates. For example, our studies have shown that compound **1** has the ability to act as a sensitive “turn‐on” sensor for trivalent metal ions, such as indium. The findings of these initial investigations also positively influence further studies on the molecular recognition of carbohydrates with fluorene‐based receptors. They provide valuable information for further structural variation of molecules with a fluorene backbone and for conducting extensive studies on the relationships between structure and binding affinity.

## Experimental Section

Melting points (uncorrected) were measured on a hot stage microscope (Büchi 510). FT‐IR spectra were obtained from a Perkin Elmer FT‐IR 1600 spectrometer as KBr pellet. ^1^H and ^13^C NMR spectra were recorded on a Bruker Avance III‐500 MHz spectrometer using Me_4_Si as internal standard. Mass spectra were recorded on a solariX 15T FT‐ICR‐MS (Bruker Daltonic). 2‐Amino‐4,6‐dimethylpyridine is commercially available. 9,9‐Diethylfluorene‐2,4,7‐tricarbaldehyde (**6**)^[10a]^ and 2,4,7‐tris(bromomethyl)‐9,9‐diethylfluorene (**7**)^[10a]^ were synthesized as described in our previous work. 2‐(*tert*‐Butyloxycarbonylamino)‐4,6‐dimethylpyridine (**8**),^[12]^ 9,9‐dihexylfluorene^[28]^ and 9,9‐didodecylfluorene^[29]^ were prepared according to the literature procedures. Spectral data of compounds **1**–**3**, **9**, **10**, **13** and **14** (NMR, IR, MS) and the syntheses of 2,4,7‐tris(bromomethyl)‐9,9‐dialkylfluorenes **11** and **12** are given in the Supporting Information.

### Synthesis of compound 1: Procedure using 9,9‐diethyl‐9H‐fluorene‐2,4,7‐tricarbaldehyde and 2‐amino‐4,6‐dimethylpyridine as reactants

**2,4,7‐Tris[(4,6‐dimethylpyridin‐2‐yl)aminomethyl]‐9,9‐diethylfluorene (1)**. In a round bottom flask, 9,9‐diethylfluorene‐2,4,7‐tricarbaldehyde (**6**, 80 mg, 0.26 mmol) and 2‐amino‐4,6‐dimethylpyridine (100 mg, 0.82 mmol) were dissolved in CH_2_Cl_2_ (5 mL) and stirred for 16 h under reflux conditions. After cooling the solution to room temperature, MeOH (5 mL) and NaBH_4_ (200 mg, 5.29 mmol) were added and the mixture stirred overnight. After removing of the solvent under reduced pressure, the residue was dissolved in CHCl_3_ (1 mL) and water (10 mL) was added. The mixture was stirred at room temperature for 4 h. The organic phase was separated from the aqueous phase and the latter extracted with CHCl_3_ (3×10 mL). The combined organic layers were dried over Na_2_SO_4_ and the solvent was removed. The product was isolated by column chromatography [eluent: CHCl_3_/MeOH (7n NH_3_) 100 : 1 (v/v), R_*f*_=0.35]. Yield 36 % of **1** (58 mg, 0.09 mmol); M.p. 101–103 °C.

### Synthesis of compounds 1–3, 9, 10, 13 and 14: Procedure using 2,4,7‐tris(bromomethyl)‐9,9‐dialkylfluorene (7, 11 or 12) and 2‐(tert‐butyloxycarbonylamino)‐4,6‐dimethylpyridine (8) as reactants

**2,4,7‐Tris[*N*‐(4,6‐dimethylpyridin‐2‐yl)‐*N*‐(*tert*‐butyloxycarbonyl)aminomethyl]‐9,9‐diethylfluorene (9)**. Compound **8** (1.80 g, 8.10 mmol) and 2,4,7‐tris(bromomethyl)‐9,9‐diethylfluorene (**7**, 1.23 g, 2.45 mmol) were dissolved in dry dioxane (20 mL). Finely ground basic alumina (Brockmann activity I, 3.27 g, 32.1 mmol) and KOH (1.00 g, 17.8 mmol) were suspended in the reaction solution. After stirring under reflux conditions for 16 h, the solid was filtered off and the solvent removed in vacuo. The raw product was purified via column chromatography [eluent: toluene/EtOAc 5 : 1 (v/v), R_*f*_=0.27]. Yield 39 % of **9** (887 mg, 0.96 mmol); M.p. 138–140 °C.

**4‐Bromomethyl‐2,7‐bis[*N*‐(4,6‐dimethylpyridin‐2‐yl)‐*N*‐(*tert*‐butyloxycarbonyl)aminomethyl]‐9,9‐diethylfluorene (10)**. Compound **8** (1.80 g, 8.10 mmol) and 2,4,7‐tris(bromomethyl)‐9,9‐diethylfluorene (**7**, 1.00 g, 2.00 mmol) were dissolved in dry dioxane (20 mL). Finely ground basic alumina (Brockmann activity I, 2.67 g, 26.4 mmol) and KOH (0.80 g, 14.3 mmol) were suspended in the reaction solution. After stirring at room temperature for 24 h, the solid was filtered off and the solvent removed in vacuo. The raw product was purified via column chromatography [eluent: toluene/EtOAc 5 : 1 (v/v), R_*f*_=0.41]. Yield 21 % of **10** (334 mg, 0.43 mmol); M.p. 128–130 °C.

Under these reaction conditions, 19 % of **9** (355 mg, 0.38 mmol) can also be obtained.

**2,4,7‐Tris[*N*‐(4,6‐dimethylpyridin‐2‐yl)‐*N*‐(*tert*‐butyloxycarbonyl)aminomethyl]‐9,9‐dihexylfluorene (13)**. Compound **8** (1.20 g, 5.40 mmol) and 2,4,7‐tris(bromomethyl)‐9,9‐dihexylfluorene (**11**, 1.00 g, 1.63 mmol) were dissolved in dry dioxane (20 mL). Finely ground basic alumina (Brockmann activity I, 2.20 g, 21.6 mmol) and KOH (0.68 g, 12.1 mmol) were suspended in the reaction solution. After stirring under reflux conditions for 16 h, the solid was filtered off and the solvent removed in vacuo. The raw product was purified via column chromatography [eluent: CHCl_3_/EtOAc 8 : 1 (v/v), R_*f*_=0.52]. Yield 35 % of **13** (592 mg, 0.57 mmol); M.p. 153–155 °C.

**2,4,7‐Tris[*N*‐(4,6‐dimethylpyridin‐2‐yl)‐*N*‐(*tert*‐butyloxycarbonyl)aminomethyl]‐9,9‐didodecylfluorene (14)**. Compound **8** (1.35 g, 6.07 mmol) and 2,4,7‐tris(bromomethyl)‐9,9‐didodecylfluorene (**12**, 1.40 g, 1.79 mmol) were dissolved in dry dioxane (20 mL). Finely ground basic alumina (Brockmann activity I, 2.40 g, 23.7 mmol) and KOH (0.75 g, 13.4 mmol) were suspended in the reaction solution. After stirring under reflux conditions for 24 h, the solid was filtered off and the solvent removed in vacuo. The raw product was purified via column chromatography [eluent: CHCl_3_/EtOAc 10 : 1 (v/v), R_*f*_=0.39]. Yield 40 % of **14** (860 mg, 0.71 mmol).

**2,4,7‐Tris[(4,6‐dimethylpyridin‐2‐yl)aminomethyl]‐9,9‐diethylfluorene (1)**. Compound **9** (500 mg, 0.54 mmol) was dissolved in CH_2_Cl_2_ (20 mL) and CF_3_COOH (1 mL, 1.48 g, 13.0 mmol) was added carefully. After stirring at room temperature for 24 hours, the solution was neutralized with 6 n sodium hydroxide solution under cooling. The organic phase was separated and the aqueous phase was extracted with CH_2_Cl_2_ (3×10 mL). The combined organic phases were washed with water, dried over Na_2_SO_4_ and the solvent was removed. No further purification was required. Yield 98 % of **1** (332 mg, 0.53 mmol).

**2,4,7‐Tris[(4,6‐dimethylpyridin‐2‐yl)aminomethyl]‐9,9‐dihexylfluorene (2)**. Compound **13** (300 mg, 0.29 mmol) was dissolved in CH_2_Cl_2_ (10 mL) and CF_3_COOH (1 mL, 1.48 g, 13.0 mmol) was added carefully. The reaction was further performed as described for **1**. Yield 86 % of **2** (186 mg, 0.25 mmol); M.p. 147–148 °C.

**2,4,7‐Tris[(4,6‐dimethylpyridin‐2‐yl)aminomethyl]‐9,9‐didodecylfluorene (3)**. Compound **14** (250 mg, 0.21 mmol) was dissolved in CH_2_Cl_2_ (15 mL) and CF_3_COOH (1 mL, 1.48 g, 13.0 mmol) was added carefully. The reaction was further performed as described for **1**. Yield 93 % of **3** (174 mg, 0.19 mmol); M.p. 48–50 °C.

### X‐ray crystallography

The data sets for the structures **1 a** and **9** were collected at temperatures of 145 and 135 K on a STOE diffractometer (MoKα radiation, λ=0.71073 Å) equipped with an image plate detector (IPDS‐2T). Indexing and integration of the reflexes were performed using the IPDS software in the X‐Area program suite.^[30]^ Data reduction and absorption correction were performed using the X‐RED program.^[30]^ Data recording for structure **10** was carried out at 153 K on a Kappa APEX II diffractometer (Bruker AXS) using MoKα radiation (λ=0.71073 Å). Including all observed reflexes, cell parameters were refined using the SAINT program.^[31]^ A semi‐empirical absorption correction was performed using SADABS.^[31]^ Preliminary structure models were created using direct methods.^[32]^ The structures were refined by full‐matrix least‐squares calculation based on *F* ^2^ values for all reflexes.^[33]^ All non‐hydrogen atoms were refined anisotropically. The hydrogen atoms H1A and H1B of the water molecule O1 in structure**1a** are located in a difference‐Fourier map. The free refinement of these H atoms however proved to be difficult which is reflected by large values of their isotropic displacement parameters. Nevertheless, the positions of these H‐atoms lead to reasonable H‐bond geometries. All other H atoms are included in the structure models and placed in calculated positions and were refined as constrained to bonding atoms.

The graphical representation of the molecular structures was performed using the program ORTEP‐III.^[34]^

Deposition Numbers 2224365 (for **1 a**), 2224363 (for **9**) and 2224364 (for **10**) contain the supplementary crystallographic data for this paper. These data are provided free of charge by the joint Cambridge Crystallographic Data Centre and Fachinformationszentrum Karlsruhe Access Structures service.

## Supporting Information Summary

Spectral data of compounds **1**–**3**, **9**, **10**, **13** and **14** (NMR, IR, MS). Syntheses of 2,4,7‐tris(bromomethyl)‐9,9‐dialkylfluorenes **11** and **12**.

Crystallographic and structure refinement data (Table S1), selected torsion angles (Table S2) and geometric parameters for selected intermolecular interactions (Table S3) in the crystal structures of the compounds **1** (as hydrate), **9** and **10**. Packing diagram of compound 9 viewed down the a‐axis (Figure S1). Views of the superpositions of **1**&**9** and **9**&**10** (Figure S2).

UV/Vis and fluorescence measurements with compounds **1**, **4** and **5** as well as some binding studies of **1** and **3** with indium ions (Figures S3–S6). Molecular recognition of carbohydrates (Figures S7–S10). ^1^H und ^13^C NMR spectra of compounds **1**–**3**, **9**, **10** and **12**–**14** (Figures S11–S26). Description of the performance of fluorescence measurements (Tables S4–S6) and ^1^H NMR titrations (Table S4).

## Conflict of interest

The authors declare no conflict of interest.

1

## Supporting information

As a service to our authors and readers, this journal provides supporting information supplied by the authors. Such materials are peer reviewed and may be re‐organized for online delivery, but are not copy‐edited or typeset. Technical support issues arising from supporting information (other than missing files) should be addressed to the authors.

Supporting Information

## Data Availability

The data that support the findings of this study are available in the supplementary material of this article.
