# Supplementary material for: Compounds Derived from 9,9‐Dialkylfluorenes: Syntheses, Crystal Structures and Initial Binding Studies (Part II)
Source: ChemistryOpen. 2023 Jul 13;12(7):e202300019. doi: 10.1002/open.202300019 (PMC10344870; doi:10.1002/open.202300019)
Supplement: Supplementary file 1 — Supporting Information [file OPEN-12-e202300019-s001.pdf]

ChemistryOpen

Supporting Information

Compounds Derived from 9,9-Dialkylfluorenes: Syntheses, Crystal Structures and Initial Binding Studies (Part II)

Pierre Seidel, Wilhelm Seichter, and Monika Mazik*

1. Spectral data of compounds **1-3**, **9**, **10**, **13** and **14**.

2. Syntheses of 2,4,7-tris(bromomethyl)-9,9-dialkylfluorenes **11** and **12**.

3. Crystallographic data (Tables S1-S3, Figures S1 and S2).

4. UV/Vis and fluorescence measurements with compounds **1**, **4** and **5** as well as some binding studies of **1** and **3** with indium ions (Figures S3-S6).

5. Molecular recognition of carbohydrates: additional information (Figures S7-S10).

6. ^{1}H und ^{13}C NMR spectra of compounds **1-3, 9**, **10** and **12-14** (Figures S11-S26).

7. Description of the performance of ^{1}H NMR and fluorescence titrations (Tables S4-S7).

1. Spectral data of compounds 1-3, 9, 10, 13 and 14

2,4,7-Tris[(4,6-dimethylpyridin-2-yl)aminomethyl]-9,9-diethylfluorene (1). ^{1}H NMR (500 MHz, $CDCl_3$): δ = 0.23 (t, *J* = 7.3 Hz, 6H), 1.97 (q, *J* = 7.3 Hz, 4H), 2.15 (s, 3H), 2.16 (s, 3H), 2.20 (s, 3H), 2.34 (s+s, 6H), 2.35 (s, 3H), 4.45 (d, *J* = 5.8 Hz, 2H), 4.48 (d, *J* = 5.8 Hz, 2H), 4.73 (t, *J* = 5.8 Hz, 1H), 4.76 (d, *J* = 5.8 Hz, 2H), 4.85 (t, *J* = 5.8 Hz, 1H), 4.88 (t, *J* = 5.8 Hz, 1H), 6.01 (s, 1H), 6.03 (s, 1H), 6.08 (s, 1H), 6.33 (s+s, 2H), 6.37 (s, 1H), 7.25 - 7.28 (m, 2H), 7.30 (d, *J* = 1.5 Hz, 1H), 7.33 (d, *J* = 1.6 Hz, 1H), 7.66 (d, *J* = 8.0 Hz, 1H) ppm. ^{13}C NMR (125 MHz, $CDCl_3$): δ = 8.4, 21.1 (2C), 21.2, 24.1 (2C), 24.2, 33.0, 45.6, 47.0, 47.1, 55.6, 103.3, 103.4 (2C), 114.2 (2C), 114.3, 121.0, 121.8, 123.2, 126.4, 126.9, 133.1, 137.7, 138.0, 138.4, 140.2, 149.0 (3C), 151.0, 151.6, 156.6 (2C), 156.7, 158.5, 158.6, 158.7 ppm. IR (KBr): ῡ = 3415, 3241, 3043, 2958, 2915, 2873, 2849, 1608, 1567, 1504, 1454, 1373, 1328, 1259, 1220, 1162, 1103, 1029, 981, 952, 923, 862, 811, 749, 663, 617, 536, 516 cm^{-1}. HRMS-ESI: $C_{41}H_{48}N_6$ calcd. for $[M + H]^+$: 625.401322, found: 625.401331.

2,4,7-Tris[(4,6-dimethylpyridin-2-yl)aminomethyl]-9,9-dihexylfluorene (2). ^{1}H NMR (500 MHz, $CDCl_3$): δ = 0.46 - 0.55 (m, 4H), 0.77 (t, *J* = 7.3 Hz, 6H), 0.95 - 1.03 (m, 8H), 1.06 - 1.14 (m, 4H), 1.86 - 1.96 (m, 4H), 2.13 (s, 3H), 2.14 (s, 3H), 2.19 (s, 3H), 2.33 (s, 3H), 2.34 (s, 3H), 2.35 (s, 3H), 4.46 (d, *J* = 5.8 Hz, 2H), 4.48 (d, *J* = 5.8 Hz, 2H), 4.75 (d, *J* = 5.8 Hz, 2H), 4.86 (t, *J* = 5.8 Hz, 1H), 5.04 (t, *J* = 5.8 Hz, 1H), 5.08 (t, *J* = 5.8 Hz, 1H), 5.98 (s, 1H), 6.00 (s, 1H), 6.07 (s, 1H), 6.31 (s, 1H), 6.32 (s, 1H), 6.36 (s, 1H), 7.25 (dd, *J* = 8.0/1.6 Hz 1H), 7.26 - 7.27 (m, 1H), 7.28 - 7.30 (m, 1H), 7.32 - 7.34 (m, 1H), 7.65 (d, *J* = 8.0 Hz, 1H) ppm. ^{13}C NMR (125 MHz, $CDCl_3$): δ = 14.0, 21.1 (2C), 21.2, 22.6, 23.7, 24.0 (2C), 24.1, 29.7, 31.5, 40.6, 45.6, 46.9, 47.0, 54.5, 103.3, 103.4 (2C), 114.2 (3C), 120.8, 121.5, 123.2, 126.2, 126.6, 133.0, 137.7, 137.9, 138.0, 139.7, 149.0 (2C), 149.1, 151.8, 152.4, 156.5 (2C), 156.7, 158.5, 158.6, 158.7 ppm. IR (KBr): ῡ = 3413, 3241, 3043, 2952, 2923, 1164, 1134, 1103, 1029, 981, 954, 923, 862, 811, 746, 667, 617, 536, 515 cm^{-1}. HRMS-ESI: $C_{49}H_{64}N_6$ calcd. for $[M + H]^+$: 737.526523, found: 737.526489.

2,4,7-Tris[(4,6-dimethylpyridin-2-yl)aminomethyl]-9,9-didodecylfluorene (3). ^{1}H NMR (500 MHz, $CDCl_3$): δ = 0.45 - 0.55 (m, 4H), 0.86 (t, *J* = 7.0 Hz, 6H), 0.95 - 1.24 (m, 32H), 1.24 - 1.30 (m, 4H), 1.85 - 1.91 (m, 4H), 2.14 (s, 3H), 2.15 (s, 3H), 2.20 (s, 3H), 2.34 (s, 3H), 2.35 (s+s, 6H), 4.46 (d, *J* = 5.7 Hz, 2H), 4.48 (d, *J* = 5.7 Hz, 2H), 4.75 (s, 3H), 4.89 (t, *J* = 5.7 Hz, 1H), 4.92 (t, *J* = 5.7 Hz, 1H), 5.99 (s, 1H), 6.01 (s, 1H), 6.08 (s, 1H), 6.32 (s+s, 2H), 6.37 (s, 1H), 7.23 - 7.27 (m, 2H), 7.27 - 7.29 (m, 1H), 7.31 - 7.33 (m, 1H), 7.65 (d, *J* = 7.9 Hz, 1H) ppm. ^{13}C NMR (125

MHz, $CDCl_3$): δ = 14.1, 21.2 (3C), 22.7, 23.8, 24.1 (2C), 24.2, 29.4 (2C), 29.6, 29.7 (3C), 30.1, 31.9, 40.6, 45.6, 47.0 (2C), 54.5, 103.3, 103.4 (2C), 114.2 (2C), 114.3, 120.8, 121.6, 123.2, 126.2, 126.7, 133.0, 137.6, 137.9 (2C), 139.7, 148.9 (2C), 149.0, 151.8, 152.4, 156.6 (2C), 156.7, 158.5, 158.6, 158.7 ppm. IR (KBr): ῡ = 3409, 3247, 3044, 2921, 2850, 1608, 1571, 1506, 1461, 1371, 1330, 1278, 1220, 1209, 1164, 1141, 1139, 1106, 1029, 981, 953, 923, 852, 811, 757, 721, 674, 617, 536, 514, 455 cm^{-1}. HRMS-ESI: $C_{61}H_{88}N_6$ calcd. for $[M + H]^+$: 905.714323, found: 905.714322.

2,4,7-Tris[*N*-(4,6-dimethylpyridin-2-yl)-*N*-(*tert*-butyloxycarbonyl)aminomethyl]-9,9-diethylfluorene (9). ^1H NMR (500 MHz, $CDCl_3$): δ = 0.12 (t, *J* = 7.3 Hz, 6H), 1.29 (s, 9H), 1.35 (s, 9H), 1.44 (s, 9H), 1.84 - 1.92 (m, 4H), 2.18 (s, 3H), 2.22 (s, 3H), 2.25 (s, 3H), 2.29 (s, 3H), 2.38 (s, 3H), 2.44 (s, 3H), 5.12 (s, 2H), 5.19 (s, 2H), 5.55 (s, 2H), 6.61 (s, 1H), 6.66 (s, 1H), 6.70 (s, 1H), 7.01 (s, 1H), 7.04 (s, 1H), 7.10 (s, 1H), 7.14 (s, 1H), 7.18 (s, 1H), 7.22 - 7.23 (m, 1H), 7.23 - 7.26 (m, 1H), 7.75 (d, *J* = 8.0 Hz, 1H) ppm. ^{13}C NMR (125 MHz, $CDCl_3$): δ = 8.3, 20.9 (2C), 21.0, 23.9, 24.0, 24.1, 28.1, 28.2, 28.3, 33.1, 48.4, 50.5, 50.6, 55.1, 80.6 (2C), 80.8, 117.7, 117.8, 117.9, 120.1, 120.3, 120.4, 120.5, 122.1, 122.8, 124.4, 126.3, 133.8, 137.0, 137.6, 137.9, 140.5, 148.2, 148.3 (2C), 150.0, 150.1 153.6, 153.8, 153.9, 154.3, 154.4 (2C), 156.2 (2C), 156.3 ppm. IR (KBr): ῡ = 2966, 2929, 2874, 2850, 1703, 1606, 1572, 1453, 1407, 1365, 1308, 1245, 1221, 1155, 1099, 1035, 987, 953, 891, 840, 768, 748, 702, 656, 616, 534, 492, 463 cm^{-1}. HRMS-ESI: $C_{56}H_{72}N_6O_6$ calcd. for $[M + H]^+$: 925.558611, found: 925.558599.

4-Bromomethyl-2,7-bis[*N*-(4,6-dimethylpyridin-2-yl)-*N*-(*tert*-butyloxycarbonyl)amino-methyl]-9,9-diethylfluorene (10). ^1H NMR (500 MHz, $CDCl_3$): δ = 0.18 (t, *J* = 7.3 Hz, 6H), 1.41 (s, 9H), 1.44 (s, 9H), 1.90 (q, *J* = 7.3 Hz, 4H), 2.26 (s+s, 6H), 2.43 (s, 6H), 4.82 (s, 2H), 5.14 (s, 2H), 5.21 (s, 2H), 6.71 (s+s, 2 H), 7.17 - 7.18 (m, 1H), 7.20 (s, 2H), 7.21 - 7.22 (m, 1H), 7.25 (s, 1H), 7.27 - 7.30 (m, 1H), 7.78 (d, *J* = 8.0 Hz, 1H) ppm. ^{13}C NMR (125 MHz, $CDCl_3$): δ = 8.3, 21.0 (2C), 24.1 (2C), 28.3 (2C), 32.8, 33.0, 50.4, 50.5, 55.3, 80.9, 81.0, 117.5, 117.7, 120.4 (2C), 122.3, 122.7, 123.1, 126.6, 128.7, 131.2, 138.3, 138.5 (2C), 139.1, 148.4 (2C), 150.4, 151.4, 153.8 (2C), 154.4 (2C), 156.3 (2C) ppm. IR (KBr): ῡ = 2968, 2924, 2875, 2852, 1704, 1606, 1571, 1449, 1408, 1365, 1307, 1245, 1221, 1156, 1100, 1034, 987, 952, 892, 841, 769, 748, 709, 657, 573, 534, 494, 466 cm^{-1}. HRMS-ESI: $C_{44}H_{55}BrN_4O_4$ calcd. for $[M + H]^+$: 783.347945, found: 783.347927.

Under these reaction conditions, 19% of **9** (355 mg, 0.38 mmol) can also be obtained.

2,4,7-Tris[*N*-(4,6-dimethylpyridin-2-yl)-*N*-(*tert*-butyloxycarbonyl)aminomethyl]-9,9-dihexylfluorene (13). ^{1}H NMR (500 MHz, $CDCl_3$): δ = 0.36 - 0.46 (m, 4H), 0.75 (t, *J* = 7.3 Hz, 6H), 0.87 - 0.99 (m, 8H), 1.04 - 1.11 (m, 4H), 1.25 (s, 9H), 1.35 (s, 9H), 1.44 (s, 9H), 1.77 - 1.86 (m, 4H), 2.23 (s, 3H), 2.24 (s, 3H), 2.26 (s, 3H), 2.30 (s, 3H), 2.38 (s, 3H), 2.44 (s, 3H), 5.14 (s, 2H), 5.21 (s, 2H), 5.57 (s, 2H), 6.64 (s, 1H), 6.66 (s, 1H), 6.71 (s, 1H), 7.01 (s, 1H), 7.04 (s, 1H), 7.17 (s, 1H), 7.20 - 7.23 (m, 3H), 7.25 - 7.26 (m, 1H), 7.73 (d, *J* = 8.0 Hz, 1 H) ppm. ^{13}C NMR (125 MHz, $CDCl_3$): δ = 14.0, 21.0 (3C), 22.7, 23.6, 24.0 (2C), 24.1, 28.1, 28.2, 28.3, 29.8, 31.6, 41.0, 48.2, 50.4 (2C), 54.1, 80.6 (2C), 80.8, 117.4, 117.5 (2C), 119.7, 120.2, 120.3 (2C), 121.9, 122.8, 123.5, 126.1, 134.2, 136.3, 137.6, 138.0, 140.1, 148.3 (3C), 150.9, 151.0, 153.7, 153.8 (2C), 154.3 (2C), 154.4, 156.1, 156.2 (2C) ppm. IR (KBr): $\bar{\upsilon}$ = 2956, 2927, 2856, 1704, 1606, 1572, 1452, 1407, 1363, 1309, 1245, 1221, 1155, 1099, 1033, 987, 953, 893, 839, 768, 704, 660, 613, 534, 492, 463 cm^{-1}. HRMS-ESI: $C_{64}H_{88}N_6O_6$ calcd. for $[M + H]^+$: 1037.683811, found: 1037.683675.

2,4,7-Tris[*N*-(4,6-dimethylpyridin-2-yl)-*N*-(*tert*-butyloxycarbonyl)aminomethyl]-9,9-didodecylfluorene (14). ^{1}H NMR (500 MHz, $CDCl_3$): δ = 0.36 - 0.46 (m, 4H), 0.86 (t, *J* = 7.0 Hz, 6H), 0.88 - 1.28 (m, 45H), 1.34 (s, 9H), 1.44 (s, 9H), 1.76 - 1.86 (m, 4H), 2.23 (s, 6H), 2.26 (s, 3H), 2.30 (s, 3H), 2.37 (s, 3H), 2.44 (s, 3H), 5.14 (s, 2H), 5.20 (s, 2H), 5.56 (s, 2H), 6.64 (s, 1H), 6.65 (s, 1H), 6.70 (s, 1H), 7.01 (s, 1H), 7.03 (s, 1H), 7.17 (s, 1H), 7.19 - 7.23 (m, 3H), 7.25 (s, 1H), 7.72 (d, *J* = 8.0 Hz, 1H) ppm. ^{13}C NMR (125 MHz, $CDCl_3$): δ = 14.1, 21.0 (3C), 22.7, 23.8, 24.0 (3C), 28.1, 28.2, 28.3, 29.3, 29.5, 29.6 (3C), 29.8, 30.3, 31.9, 41.0, 48.2, 50.4 (2C), 54.1, 80.6 (2C), 80.8, 117.4, 117.5 (2C), 119.7, 120.2, 120.3 (2C), 121.9, 122.8, 123.5, 126.1, 134.2, 136.4, 137.6, 138.0, 140.1, 148.3 (3C), 150.9, 151.0, 153.7, 153.8, 153.9, 154.3 (2C), 154.4, 156.1, 156.2 (2C) ppm. IR (KBr): $\bar{\upsilon}$ = 2954, 2923, 2852, 1708, 1606, 1571, 1454, 1407, 1365, 1307, 1245, 1220, 1155, 1099, 1033, 1000, 987, 952, 923, 892, 858, 838, 769, 719, 703, 659, 638, 615, 561, 534, 491, 462 cm^{-1}. HRMS-ESI: $C_{76}H_{112}N_6O_6$ calcd. for $[M + H]^+$: 1205.871612, found: 1205.871612.

2. Syntheses of 2,4,7-tris(bromomethyl)-9,9-dialkylfluorenes 11 and 12

2,4,7-Tris(bromomethyl)-9,9-dihexylfluorene (11). In a round-bottom flask 9,9-dihexylfluorene[28] (10.0 g, 30.0 mmol) was dissolved in 20 ml chloroform, paraformaldehyde (9.0 g, 300 mmol) and a 33% solution of HBr in acetic acid (80 ml) were added. The mixture was stirred under reflux conditions for 3d, cooled down and poured into water (50 ml). The opaque organic layer was separated and chloroform added to obtain a clear solution. The organic phase was washed with a saturated $NaHCO_3$ solution (2 x 20 ml) and water (2 x 20 ml), dried over Na_2SO_4, and the solvent was removed under reduced pressure. The raw product was purified via column chromatography [eluent: *n*-hexane/toluene 10:1 (*v/v*)]. Yield 59 % of **11** (10.8 g, 17.6 mmol); M.p. 43 - 45 °C. ^{1}H NMR (500 MHz, $CDCl_3$): δ = 0.52 - 0.63 (m, 4H), 0.75 (t, *J* = 7.3 Hz, 6H), 0.99 - 1.05 (m, 8H), 1.06 - 1.14 (m, 4H), 1.90 - 2.00 (m, 4H), 4.54 (s, 2 H), 4.58 (s, 2H), 4.81 (s, 2H), 7.29 (d, *J* = 1.7 Hz, 1 H), 7.33 (d, *J* = 1.7 Hz, 1 H), 7.39 (d, *J* = 1.7 Hz, 1 H), 7.41 (dd, *J* = 7.9/1.7 Hz, 1 H), 7.89 (d, *J* = 7.9 Hz, 1 H) ppm.
Reference 28 is given in the main text.

2,4,7-Tris(bromomethyl)-9,9-didodecylfluorene (12). In a round-bottom flask 9,9-didodecylfluorene[29] (20.4 g, 40.6 mmol) was dissolved in 30 ml chloroform, paraformaldehyde (12.4 g, 413 mmol) and a 33% solution of HBr in acetic acid (150 ml) were added. The mixture was refluxed for 7d, cooled down and poured into water (100 ml). The opaque organic layer was separated and chloroform added to obtain a clear solution. The organic phase was washed with a saturated $NaHCO_3$ solution (2 x 30 ml) and water (2 x 30 ml), dried over Na_2SO_4, and the solvent was removed under reduced pressure. The raw product was purified via column chromatography [eluent: n-hexane]. Yield 36 % of **12** (11.5 g, 14.7 mmol). ^{1}H NMR (500 MHz, $CDCl_3$): δ = 0.52 - 0.63 (m, 4H), 0.86 (t, *J* = 7.0 Hz, 6H), 1.00 - 1.23 (m, 32H), 1.23 - 1.31 (m, 4H), 1.91 - 1.97 (m, 4H), 4.56 (s, 2 H), 4.60 (s, 2H), 4.84 (s, 2H), 7.30 (d, *J* = 1.7 Hz, 1 H), 7.32 (d, *J* = 1.7 Hz, 1 H), 7.38 (d, *J* = 1.7 Hz, 1 H), 7.43 (dd, *J* = 8.0/1.7 Hz, 1 H), 7.89 (d, *J* = 8.0 Hz, 1 H) ppm. ^{13}C NMR (125 MHz, $CDCl_3$): δ = 14.2, 22.7, 23.6, 29.2, 29.4, 29.5 (2C), 29.6 (2C), 29.9, 31.9, 32.1, 33.4, 34.0, 40.2, 54.8, 123.6, 123.9, 124.1, 128.3, 130.0, 132.3, 136.9, 137.1, 139.0, 139.7, 152.4, 153.4 ppm. IR (KBr): $\tilde{\upsilon}$ = 2953, 2919, 2850, 1457, 1417, 1376, 1346, 1299, 1270, 1236, 1207, 1151, 1108, 1004, 980, 945, 889, 825, 788, 755, 721, 669, 653, 574, 455 cm^{-1}. HRMS-LDI: $C_{40}H_{61}Br_3$ calcd. for $[M-Br]^+$: 699.313454, found 699.313452.
References are given in the main text.

3. Crystallographic data

Table S1. Crystallographic and structure refinement data of **1a**, **9** and **10**.

Compound	**1a**	**9**	**10**
Empirical formula	$C_{41}H_{48}N_6 \cdot 2\ H_2O$	$C_{56}H_{72}N_6O_6$	$C_{33}H_{48}N_4O_4$ Br
Formula weight	660.88	925.19	783.83
Crystal system	Triclinic	Triclinic	Triclinic
Space group	*P*-1	*P*-1	*P*-1
a (Å)	11.843(4)	11.371(7)	12.5522(5)
b (Å)	13.325(3)	15.792(9)	12.6092(5)
c (Å)	14.356(4)	16.672(6)	14.8160(6)
α (°)	114.29(2)	67.14(4)	93.9244(17)
β (°)	113.64(2)	74.36(4)	105.6933(16)
γ (°)	90.67(3)	83.07(5)	109.3592(16)
V (Å^3)	1848.6(10)	2656(3)	2097.40(15)
Z	2	2	2
F(000)	712	996	828
D_c (Mg m^{-3})	1.187	1.157	1.241
μ (mm^{-1})	0.074	0.075	1.025
Data collection			
Temperature (K)	145(2)	135(2)	153(2)
No. of collected reflections	26409	22954	23999
within the θ-limit (°)	2.8 - 26.0	2.6 - 25.5	1.8 - 26.0
Index ranges ±*h*, ±*k*, ±*l*	-14/14, -16/16, -17/17	-13/13, -19/19, -20/20	-15/15, -15/15, -18/18
No. of unique reflections	7268	22954	8163
R_{int}	0.0586	0.0838	0.0333
Refinement calculations: full-matrix least- squares on all F^2 values			
Weighting expression *w* [a]	$[\sigma^2(F_o^2)+(0.0675P)^2 + 0.8436P)^{-1}$	$[\sigma^2(F_o^2)+(0.0530P)^2 + 1.7109P)^{-1}$	$[\sigma^2(F_o^2)+(0.0429P)^2 +0.8422P)^{-1}$
No. of refined parameters	478	631	490
No. of F values used [$I>2\sigma(I)$]	5723	14042	6171
Final *R*-Indices			
$R(=\Sigma\|\Delta F\| / \Sigma\|F_o\|)$	0.0483	0.0634	0.0401
wR on F^2	0.1404	0.1633	0.1015
S (=Goodness of fit on F^2)	1.025	1.040	1.146
Final $\Delta\rho_{max}/\Delta\rho_{min}$ (e Å^{-3})	0.47/-0.39	0.30/-0.33	0.38/-0.55

[a] $P = (F_o^2 + 2F_c^2)/3$

Table S2. Relevant conformational parameters of **1a**, **9** and **10**.

Compound	*1a*	*9*	10
Dihedral angles (°)[a]			
mpla(A)-mpla(B)	78.9(1)	85.8(1)	65.0(1)
mpla(A)-mpla(C)	75.4(1)	86.3(1)	59.4(1)
mpla(A)-mpla(D)	83.2(1)	76.8(1)	
mpla(B)-mpla(C)	6.5(1)	15.5(2)	57.3(1)
mpla(B)-mpla(D)	18.7(1)	12.3(2)	
mpla(C)-mpla(D)	21.5(1)	11.7(2)	
mpla(A^1)-mpla(A^2)	8.8(1)		
mpla(A^0)-mpla(E)	88.0(1)		
mpla(A)-mpla(E)		89.1(1)	85.4(1)
Torsion angles			
C(2)-C(3)-C(18)-N(1)	141.6(2)	-94.2(2)	27.8(3)
C(3)-C(18)-N(1)-C(19)	-64.4(2)	74.0(4)	-99.6(2)
C(18)-N(1)-C(19)-N(2)	174.7(1)	29.9(4)	26.8(2)
C(4)-C(5)-C(26)-N(3)	-7.7(2)		
C(5)-C(26)-N(3)-C(27)	-80.7(2)		
C(26)-N(3)-C(27)-N(4)	-170.7(1)		
C(9)-C(10)-C(34)-N(5)	-165.1(1)		
C(10)-C(34)-N(5)-C(35)	89.1(2)		
C(34)-N(5)-C(35)-N(6)	157.2(2)		
C(19)-N(1)-C(26)-O(2)		-173.1(3)	-177.3(2)
N(1)-C(26)-O(2)-C(27)		-175.6(3)	-157.1(2)
O(1)-C(26)-O(2)-C(27)		3.4(6)	24.2(3)
C(18)-N1)-C(19)-N(2)			26.8(2)
C(26)-N(1)-C(19)-N(2)			-140.3(2)
C(4)-C(3)-C(18)-N(1)			155.0(2)
C(3)-C(18)-N(1)-C(19)			-99.6(2)
C(33)-N(3)-C(40)-O(4)			175.0(2)
N(3)-C(40)-O(4)-C(41)			159.6(2)
O(3)-C(40)-O(4)-C(41)			-21.6(3)
C(32)-N(3)-C(33)-N(4)			31.1(2)
C(40)-N(3)-C(33)-N(4)			-154.2(2)
C(9)-C(10)-C(32)-N(3)			-35.4(3)
C(10)-C(32)-N(3)-C(33)			108.7(2)
C(4)-C(5)-C(31)-Br(1)			96.9(2)
C(4)-C(5)-C(31)-N(3)		-29.8(5)	
C(5)-C(31)-N(3)-C(32)		109.1(4)	
C(31)-N(3)-C(32)-N(4)		-17.2(5)	
C(32)-N(3)-C(39)-O(4)		167.3(3)	
N(3)-C(39)-O(4)-C(40)		166.0(3)	
O(3)-C(39)-O(4)-C(40)		-15.0(5)	
C(9)-C(10)-C(44)-N(5)		149.0(3)	
C(10)-C(44)-N(5)-C(45)		118.2(4)	
C(44)-N(5)-C(45)-N(6)		2.0(5)	
C(45)-N(5)-C(52)-O(6)		177.5(3)	
N(5)-C(52)-O(6)-C(53)		174.2(3)	
O(5)-C(52)-O(6)-C(53)		-7.1(7)	

[a] mpla means least-squares plane through the aromatic ring or the pyridine unit.
1a: Ring A: C(1)…C(13); ring B: N(2),C(19)…C(23); ring C: N(4),C(27)…C(31); ring D: N(6),C(35)…C(39)
9: Ring A: C(1)…C(13); ring B: N(2),C(19)…C(23); ring C: N(4),C(32)…C(36); ring D: N(6),C(45)…C(49); mpla(E): C(13)…C(17). **10**: Ring A: C(1)…C(13); ring B: N(2),C(19)…C(23); ring C: N(4),C(33)…C(37); mpla(E): C(13)…C(17).

Table S3. Non-covalent interactions in the crystals **1, 9** and **10.**

Atoms		Distance (Å)		Angle (°)	Slippage (Å)
D-H···A		D···A	H···A	D-H···A	
C-H···π		C···*Cg*	H···*Cg*	C-H···*Cg*	
π···π		*Cg*···*Cg*			
**1a**					
N(1)-H(1)···N(2)	-*x*, 1-*y*, -*z*	3.111(2)	2.22(1)	172(2)	
N(3)-H(3)···O(2)	*x*, *y*, *z*	2.924(2)	2.06(1)	162(2)	
N(5)-H(5)···O(1)	*x*, *y*, 1+*z*	3.037(2)	2.16(2)	163(2)	
O(1)-H(1A)···N(4)	1-*x*, 1-*y*, -*z*	2.823(2)	1.97(1)	177	
O(1)-H(1B)···N(1)	1+*x*, *y*, *z*	3.101(2)	2.37	141	
O(2)-H(2A)···N(6)	*x*, *y*, -1+*z*	2.859(2)	1.98(1)	178(2)	
O(2)-H(2B)···O(1)	*x*, *y*, *z*	2.812(2)	1.95(1)	165(3)	
C(24)-H(24C)···*Cg*(D)[a]	1-*x*, 1-*y*, 1-*z*	3.715(3)	2.84	149.2	
C(26)-H(26A)···*Cg*(A^2)[a]	1-*x*, 1-*y*, 1-*z*	3.694(2)	2.81	149.8	
C(32)-H(32B)···*Cg*(D)[a]	*x*, *y*, -1+*z*	3.793(3)	2.89	154.2	
C(34)-H(34A)···*Cg*(B)[a]	1-*x*, 1-*y*, 1-*z*	3.620(3)	2.72	151.4	
C(40)-H(40B)···*Cg*(C)[a]	*x*, *y*, 1+*z*	3.795(4)	2.89	153.3	
**9**					
C(4)-H(4)···N(3)	*x*, *y*, *z* (*intra*)	2.855(4)	2.53	100.3	
C(18)-H(18A)···N(2)	*x*, *y*, *z* (*intra*)	2.707(6)	2.35	100.0	
C(18)-H(18B)···O(2)	*x*, *y*, *z* (*intra*)	2.646(5)	2.22	104.3	
C(23)-H(23)···O(1)	*x*, *y*, *z* (*intra*)	2.855(6)	2.41	108.6	
C(25)-H(25A)···O(5)	*x*, 1+*y*, *z*	3.356(6)	2.57	137.5	
C(29)-H(29B)···O(1)	*x*, *y*, *z* (*intra*)	2.937(6)	2.40	114.2	
C(29)-H(29C)···O(3)	*x*, *y*, *z* (*intra*)	3.551(6)	2.57	177.4	
C(30)-H(30C)···O(1)	*x*, *y*, *z* (*intra*)	3.043(7)	2.46	117.6	
C(31)-H(31A)···N(4)	*x*, *y*, *z* (*intra*)	2.738(6)	2.32	104.4	
C(36)-H(36)···O(3)	*x*, *y*, *z* (*intra*)	2.857(6)	2.35	112.8	
C(41)-H(41C)···O(3)	*x*, *y*, *z* (*intra*)	2.979(7)	2.42	116.0	
C(44)-H(44A)···N(6)	*x*, *y*, *z* (*intra*)	2.684(6)	2.16	111.1	
C(49)-H(49)···O(5)	*x*, *y*, *z* (*intra*)	2.749(6)	2.13	121.5	
C(54)-H(54C)···O(5)	*x*, *y*, *z* (*intra*)	3.013(7)	2.42	118.8	
C(56)-H(56B)···O(5)	*x*, *y*, *z* (*intra*)	2.962(7)	2.39	117.0	
C(28)-H(28B)···*Cg*(C)[a]	*x*, *y*, *z* (*intra*)	3.848(5)	2.97	149.9	
C(44)-H(44B)···*Cg*(C)[a]	2-*x*, -*y*, -*z*	3.344(4)	2.59	133.3	
Cg(B)···*Cg*(D)[a]	1-*x*, -*y*, 1-*z*	3.922(3)			1.322
Cg(D)···*Cg*(B)[a]	1-*x*, -*y*, 1-*z*	3.922(3)			1.870
**10**					
C(29)-H(29C)···O(1)	*x*, *y*, *z* (*intra*)	3.085(4)	2.52	116.3	
C(30)-H(30B)···O(1)	*x*, *y*, *z* (*intra*)	3.065(3)	2.50	116.4	
C(31)-H(31B)···O(3)	-*x*, 1-*y*, -*z*	3.257(3)	2.36	151.2	
C(32)-H(32A)···N(4)	*x*, *y*, *z* (*intra*)	2.766(3)	2.30	107.7	
C(42)-H(42C)···O(3)	*x*, *y*, *z* (*intra*)	2.897(4)	2.37	113.3	
C(43)-H(43B)···O(3)	*x*, *y*, *z* (*intra*)	3.082(4)	2.53	115.5	
C(23)-H(23)···*Cg*(C)[a]	1+*x*, 1+*y*, 1+*z*	3.536(2)	2.75	141.0	
C(31)-H(31A)···*Cg*(B)[a]	1-*x*, 2-*y*, 1-*z*	3.726(3)	2.76	165.1	

[a] *Cg* means the centroid (centre of gravity) of the aromatic ring.
1a: Ring A^2: C(7)…C(12); ring B: N(2),C(19)…C(23); ring C: N(4),C(27)…C(31); ring D: N(6),C(35)…C(39).
9: Ring B: N(2),C(19)…C(23); ring C: N(4),C(32)…C(36); ring D: N(6), C(45)…C(49).
10: Ring B: N(2),C(19)…C(23); ring C: N(4),C(33)…C(37).

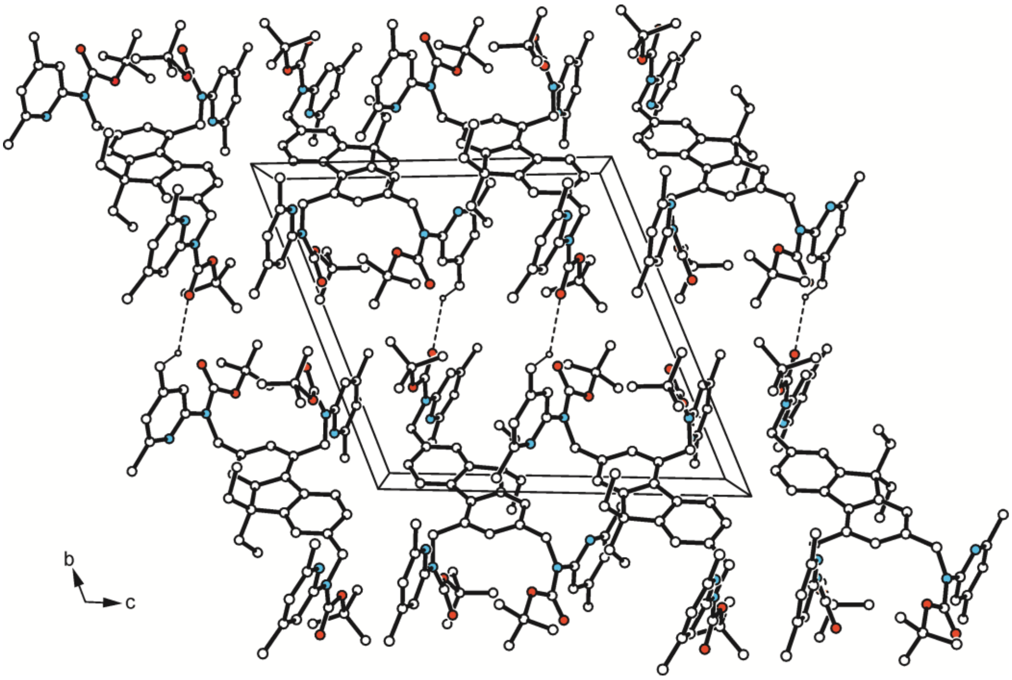

Figure S1: Packing diagram of compound **9** viewed down the *a*-axis. Broken lines represent hydrogen bonds.

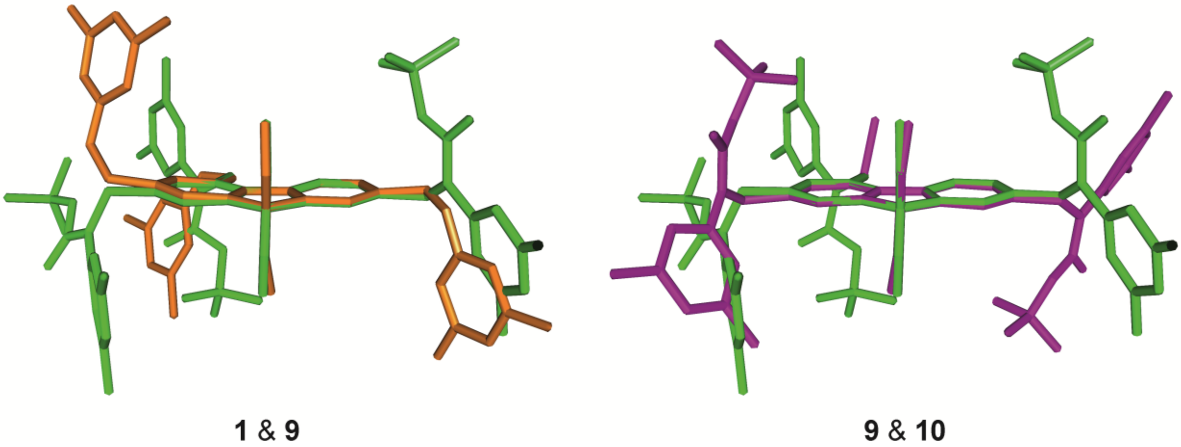

Figure S2. Views of the superpositions of **1**&**9** and **9**&**10** fitted on the atoms C6, C7 and C13 (see Figure 5) of the molecules **1** (crystal structure **1a**), **9** and **10** (H atoms are omitted for clarity). Colour code: **1**, orange; **9**, green; **10**, purple.

4. UV/Vis and fluorescence measurements with compounds 1, 4 and 5 as well as some binding studies of 1 and 3 with indium ions (Figures S3-S6)

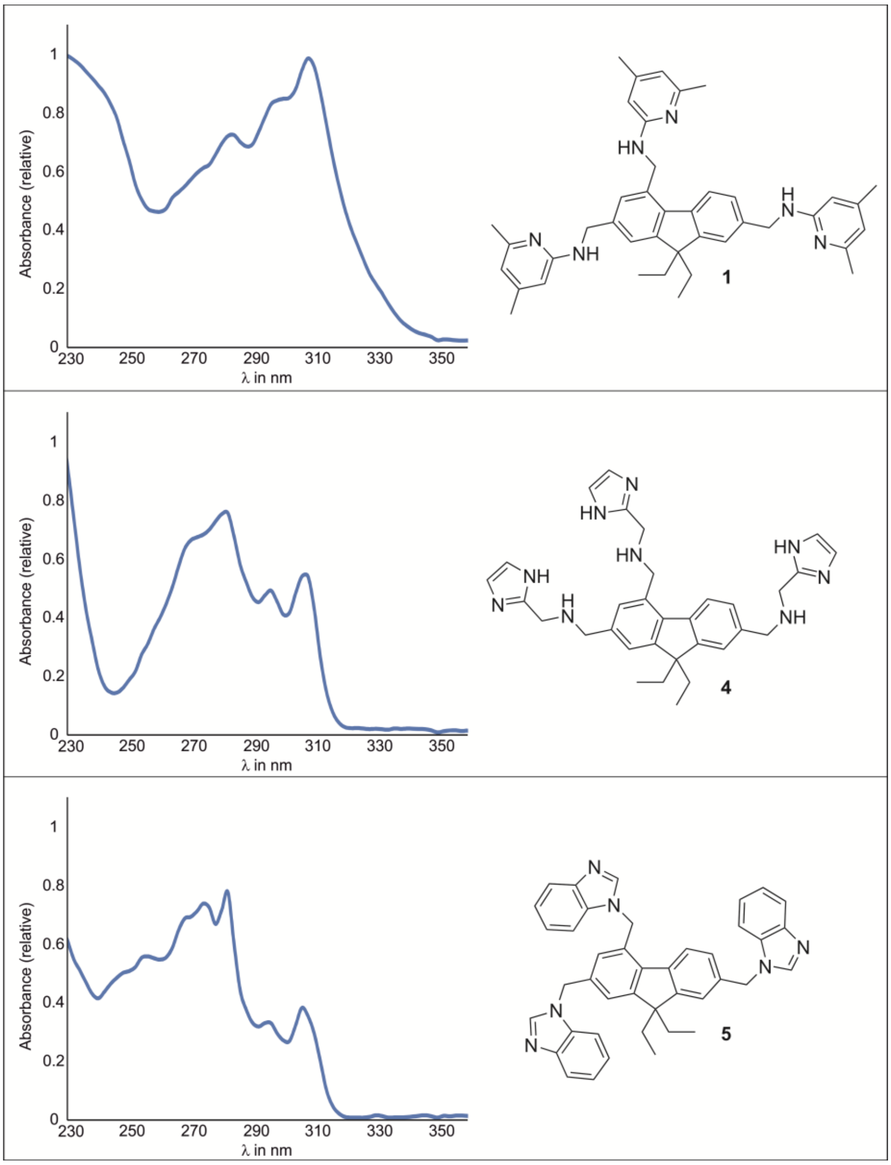

Figure S3. UV/Vis spectra of **1**, **4** and **5** (c = $1 \cdot 10^{-5}$ mol/L), relative to the spectrum of **1** (absorbance at 230 nm set to 1).

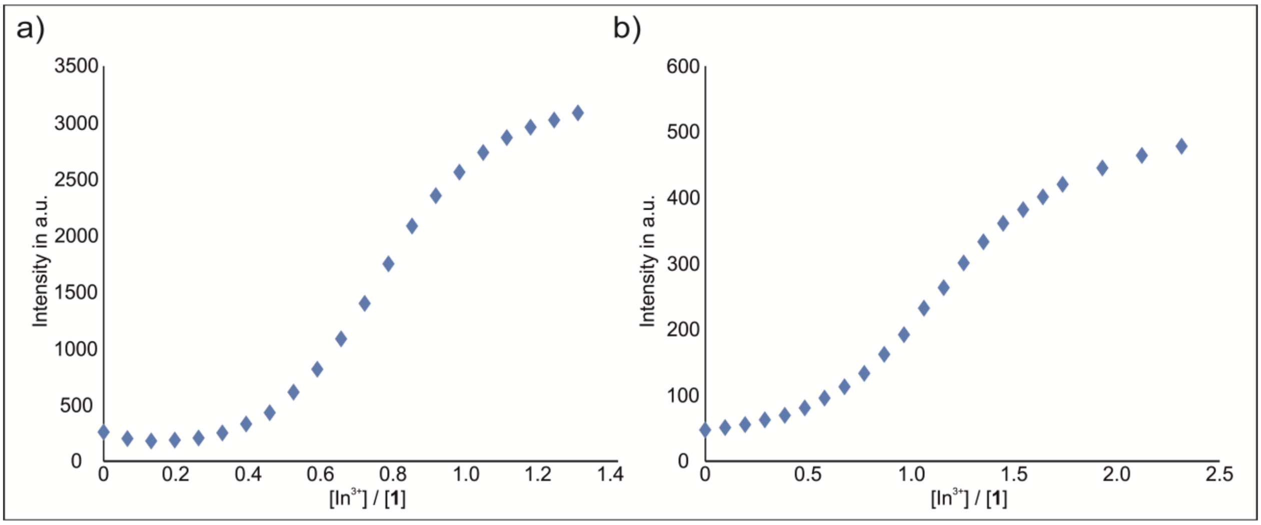

Figure S4. Results of the fluorescence titrations of **1** with $InCl_3$ presented as a plot of the emission intensity (at 380 nm) against the In^{3+}/**1** ratio at a receptor concentration of $4{\cdot}10^{-5}$ mol/L (a) and $1{\cdot}10^{-6}$ mol/L (b).

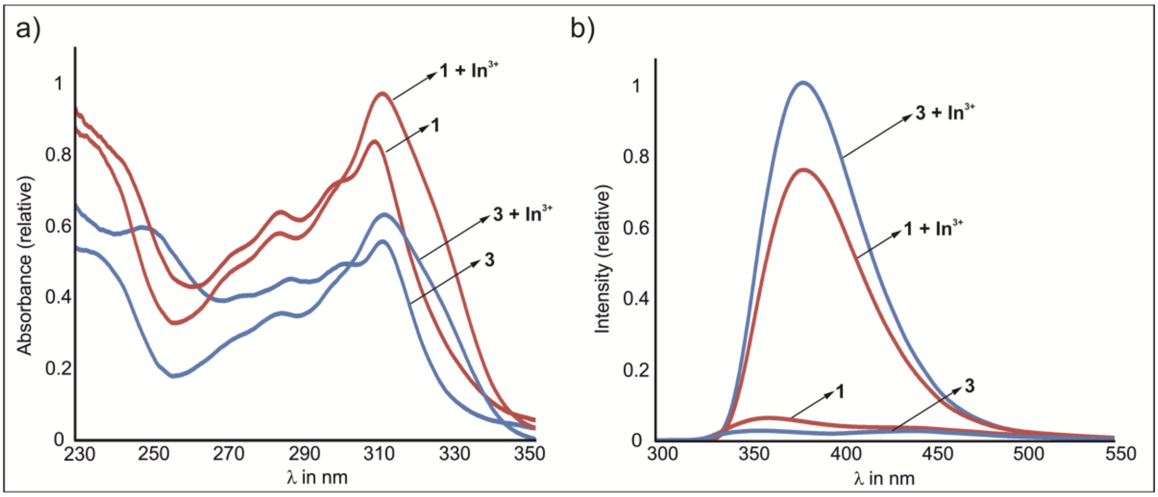

Figure S5. (a) UV/Vis spectra of **1** and **3** (c = $4{\cdot}10^{-5}$ mol/L) without and with the addition of two equivalents of $InCl_3$, relative to the spectrum of **1+In**$^{3+}$ (absorbance at 312 nm set to 1); (b) Fluorescence spectra (excitation at 250 nm) of **1** and **3** (c = $4{\cdot}10^{-5}$ mol/L) without and with the addition of two equivalents of $InCl_3$, relative to the spectrum of **3+In**$^{3+}$ (maximum set to 1).

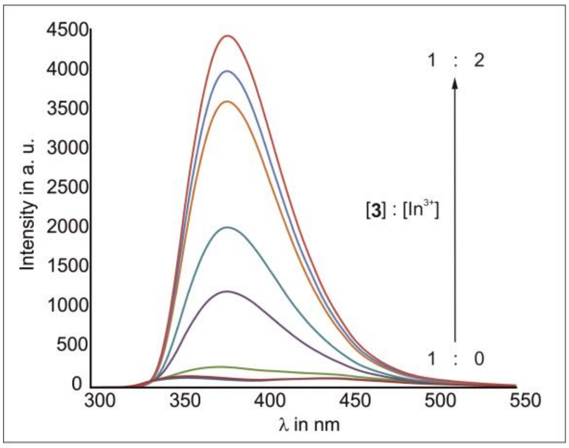

Figure S6. Fluorescence spectra of **3** (c = $4{\cdot}10^{-5}$ mol/L) upon the addition of up to two equivalents of $InCl_3$.

5. Molecular recognition of carbohydrates: additional information (Figures S7-S10)

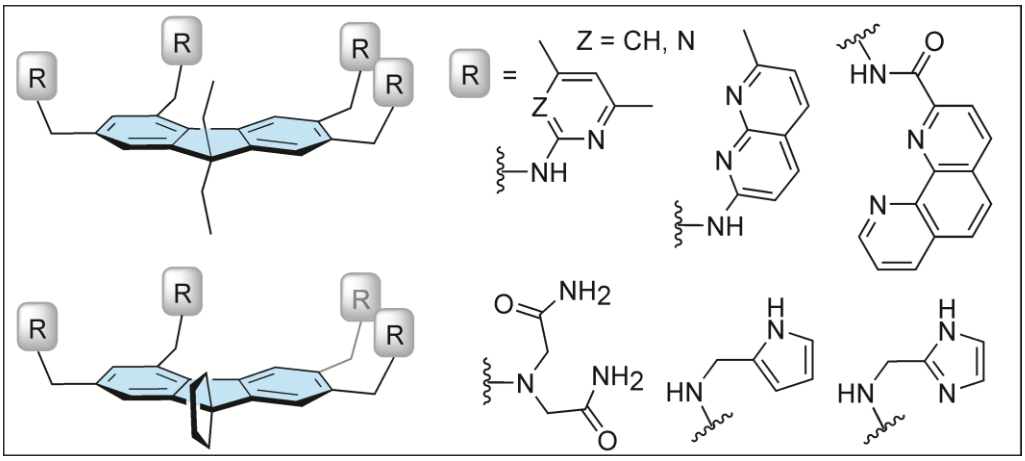

Figure S7. Examples of planned structural variations of the fluorene backbone (R = recognition units).

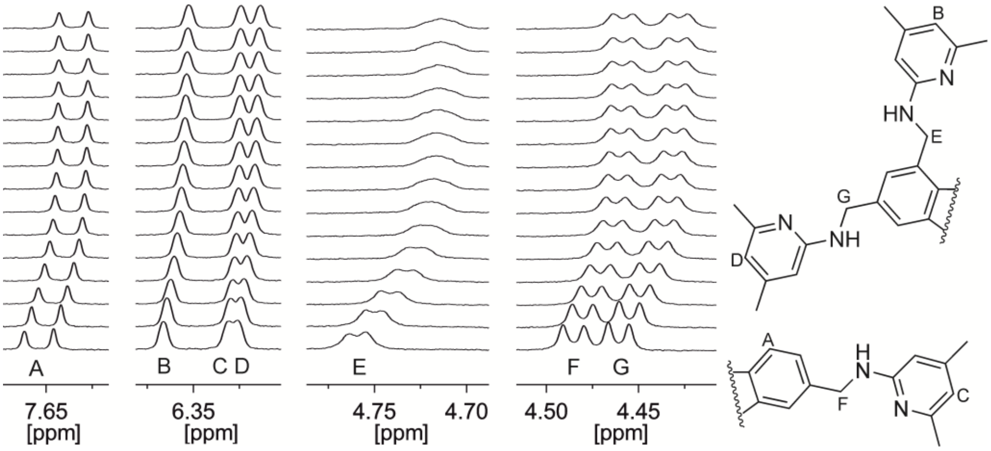

Figure S8. Excerpts of the ^{1}H NMR spectra (500 MHz, $CDCl_3$) of compound **1** (c= $1 \cdot 10^{-3}$ mol/L) after addition of (from bottom to top) 0.00-10.00 equiv of octyl β-D-glucopyranoside. Shown are the chemical shifts of the groups marked in the structural formula.

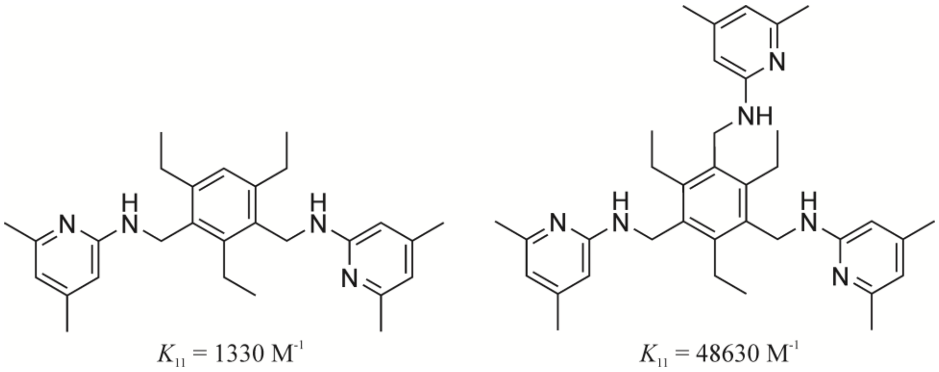

Figure S9. Structures of 1,3-bis[(4,6-dimethylpyridin-2-yl)aminomethyl]-2,4,6-triethyl-benzene[27a] and 1,3,5-tris[(4,6-dimethylpyridin-2-yl)aminomethyl]-2,4,6-triethylbenzene[27b] as well as the determined binding constants for octyl β-D-glucoside in $CDCl_3$ (References are given in the main text).

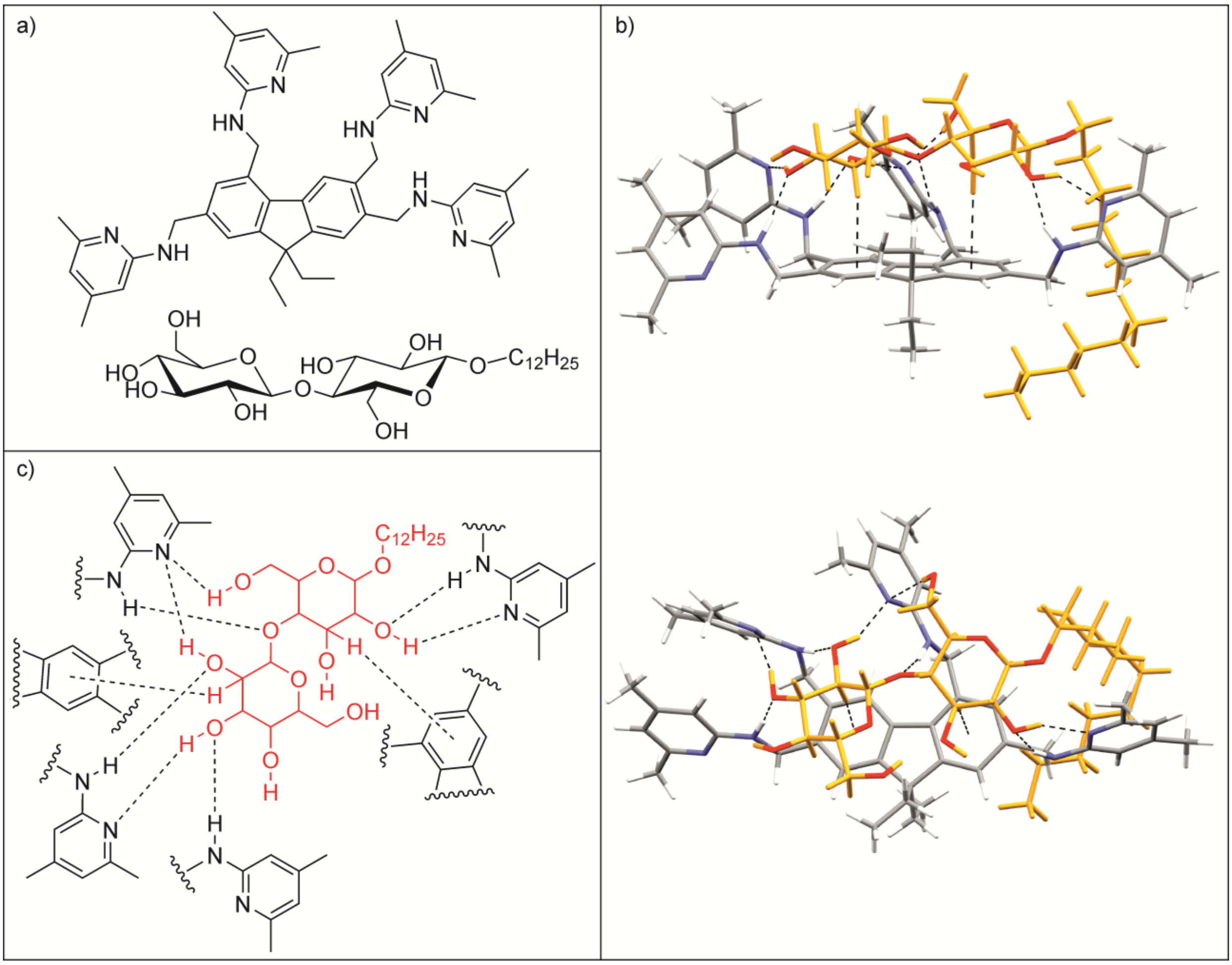

Figure S10. a) Structures of the fluorene derivate with four aminopyridine-based recognition units and of dodecyl β-D-cellobioside. b) Energy-minimized structure of the 1:1 receptor-disaccharide complex (two views; the disaccharide is located in the cavity of the receptor molecule). c) Schematic representation of the non-covalent interactions that stabilize the complex MacroModel V.9.8, OPLS_2001 force field, MCMM, 50000 steps; Color code: receptor N, blue; receptor C, grey; sugar O, red; sugar C/H, yellow.

6. ^{1}H and ^{13}C NMR spectra of compounds 1-3, 9, 10, and 12 - 14 (Figures S11-S26)

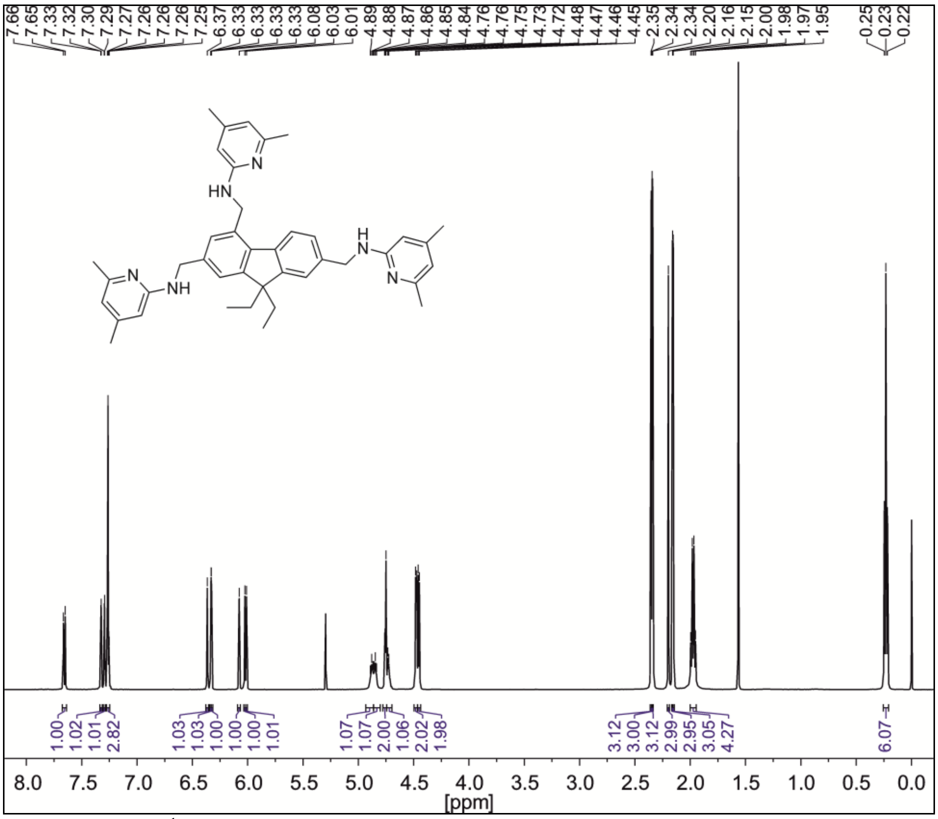

Figure S11. ^{1}H NMR (500 MHz) spectrum of **1** in $CDCl_3$.

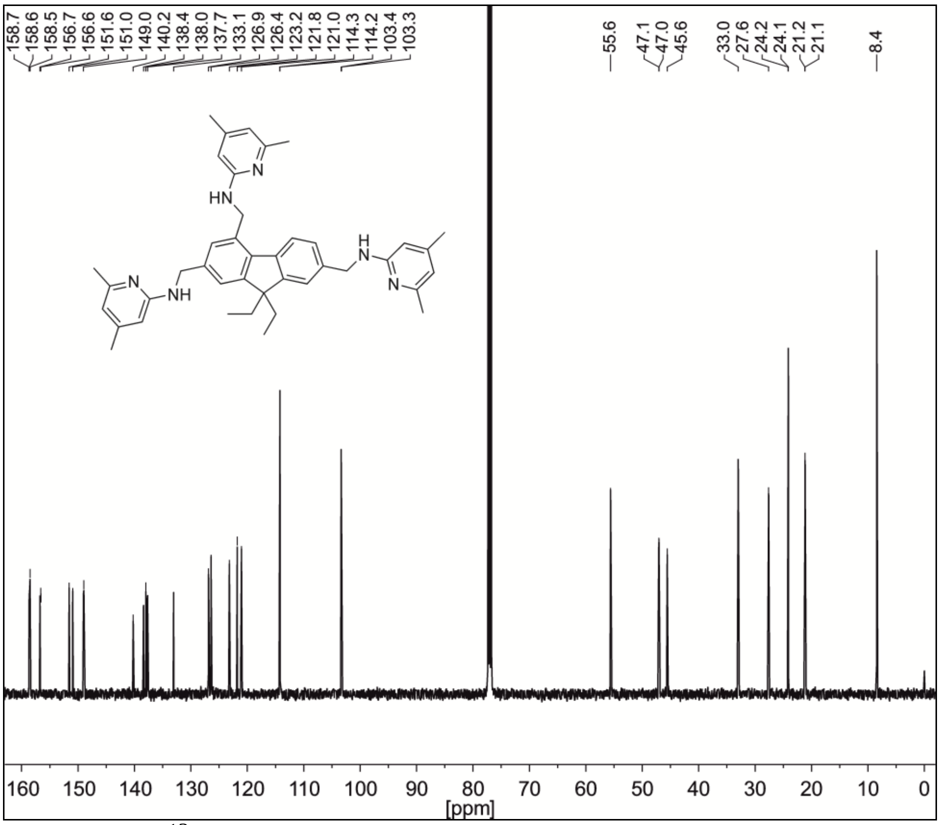

Figure S12. ^{13}C NMR (125 MHz) spectrum of **1** in $CDCl_3$.

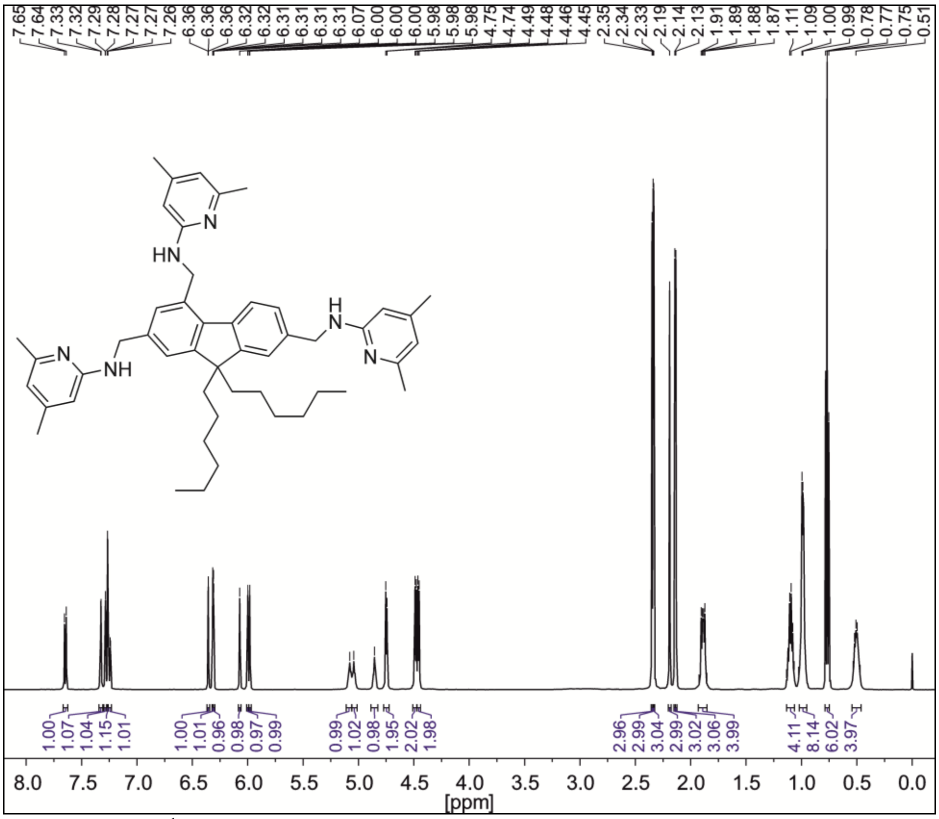

Figure S13. ^{1}H NMR (500 MHz) spectrum of **2** in $CDCl_3$.

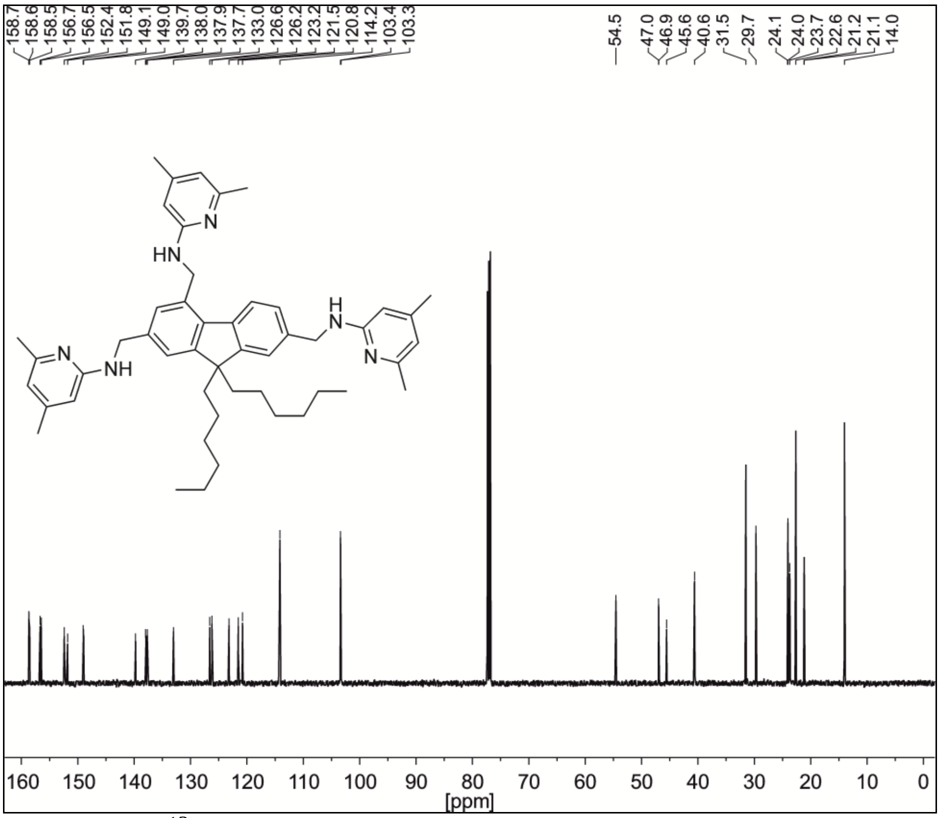

Figure S14. ^{13}C NMR (125 MHz) spectrum of **2** in $CDCl_3$.

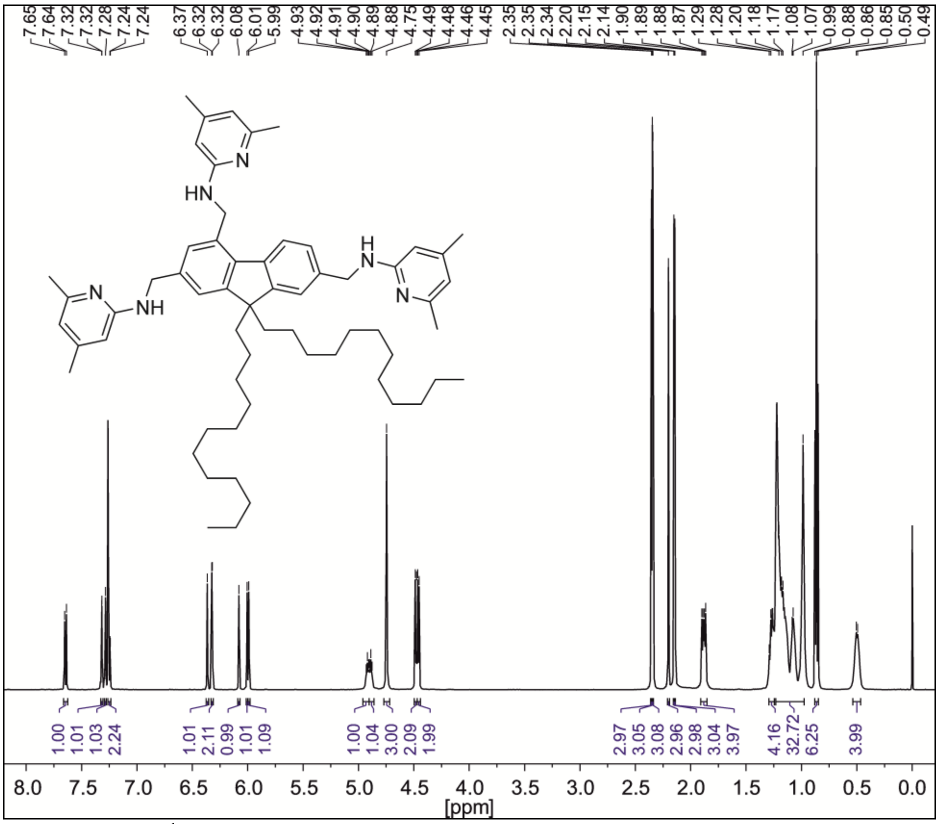

Figure S15. ^{1}H NMR (500 MHz) spectrum of **3** in $CDCl_3$.

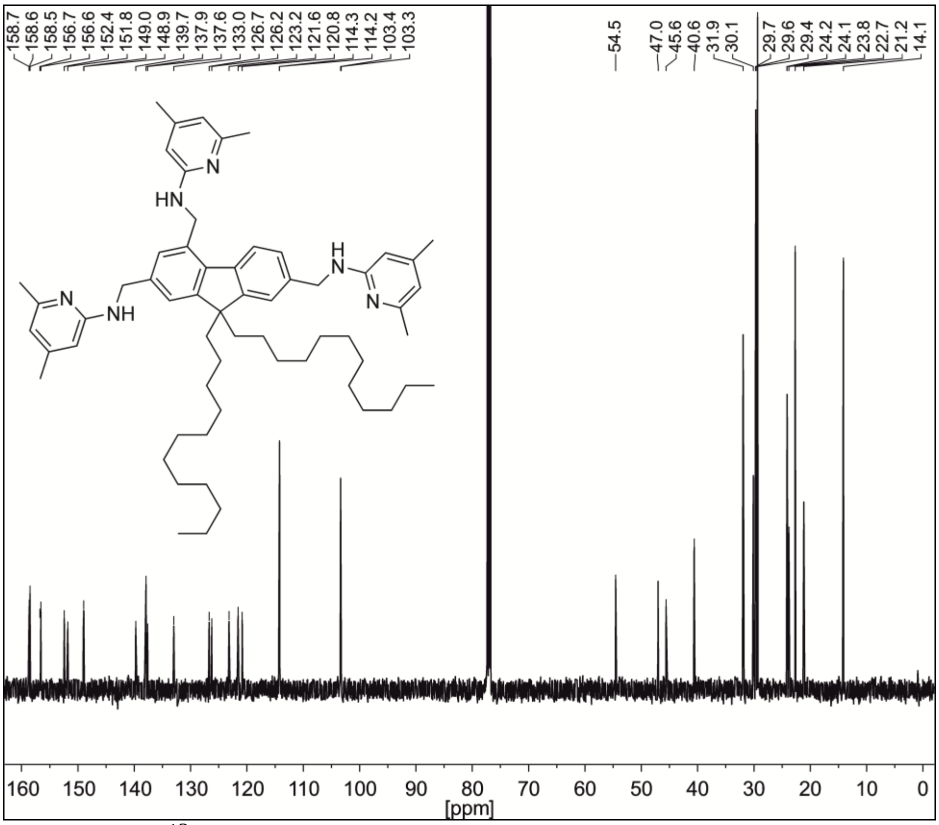

Figure S16. ^{13}C NMR (125 MHz) spectrum of **3** in $CDCl_3$.

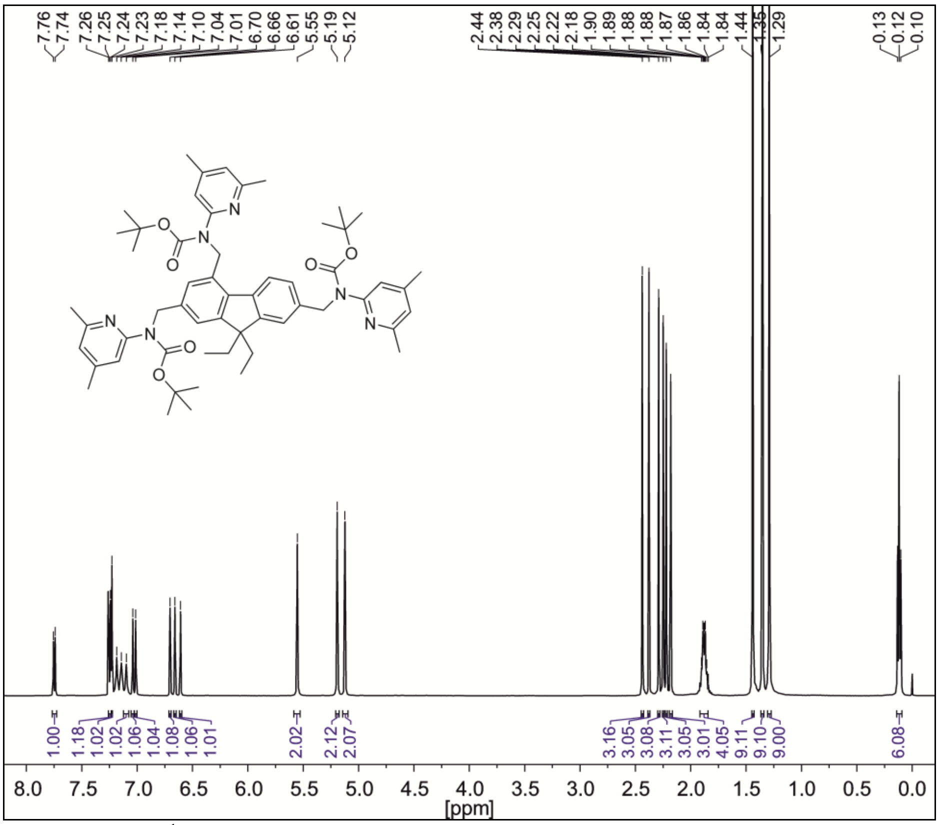

Figure S17. ^{1}H NMR (500 MHz) spectrum of **9** in $CDCl_3$.

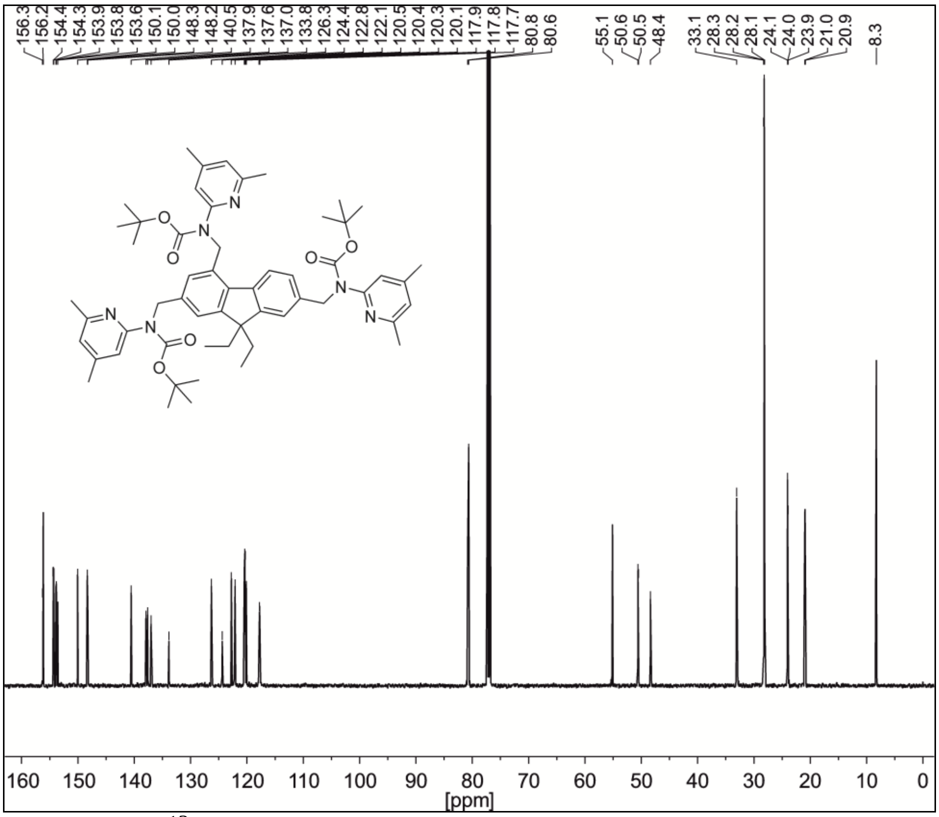

Figure S18. ^{13}C NMR (125 MHz) spectrum of **9** in $CDCl_3$.

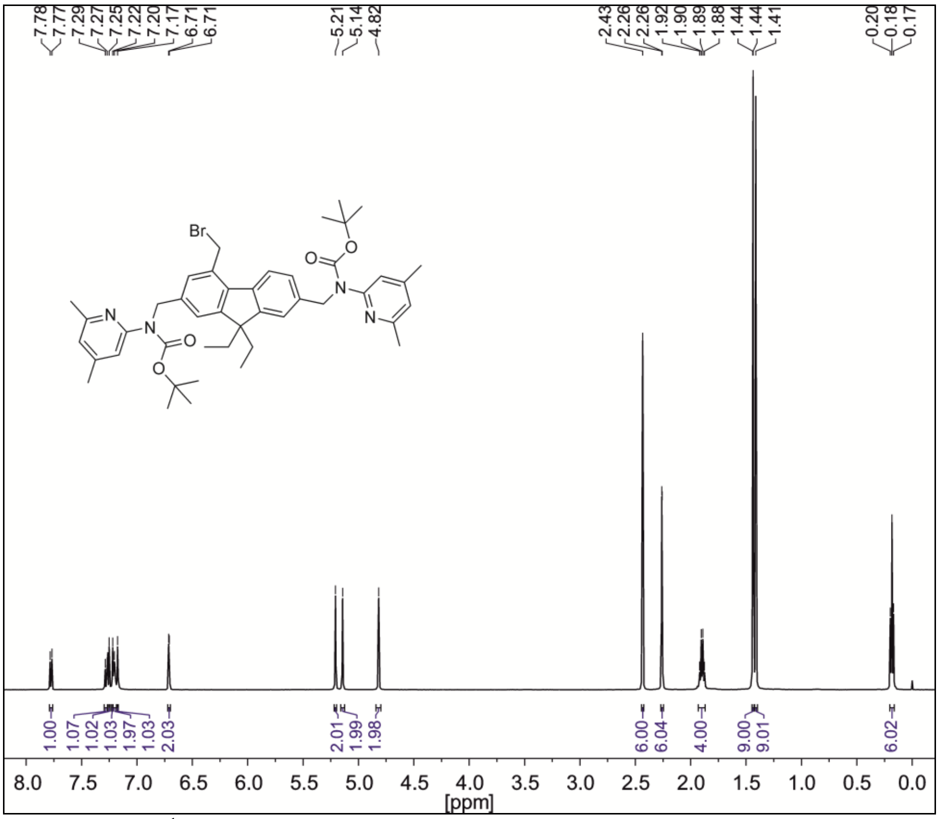

Figure S19. ^{1}H NMR (500 MHz) spectrum of **10** in $CDCl_3$.

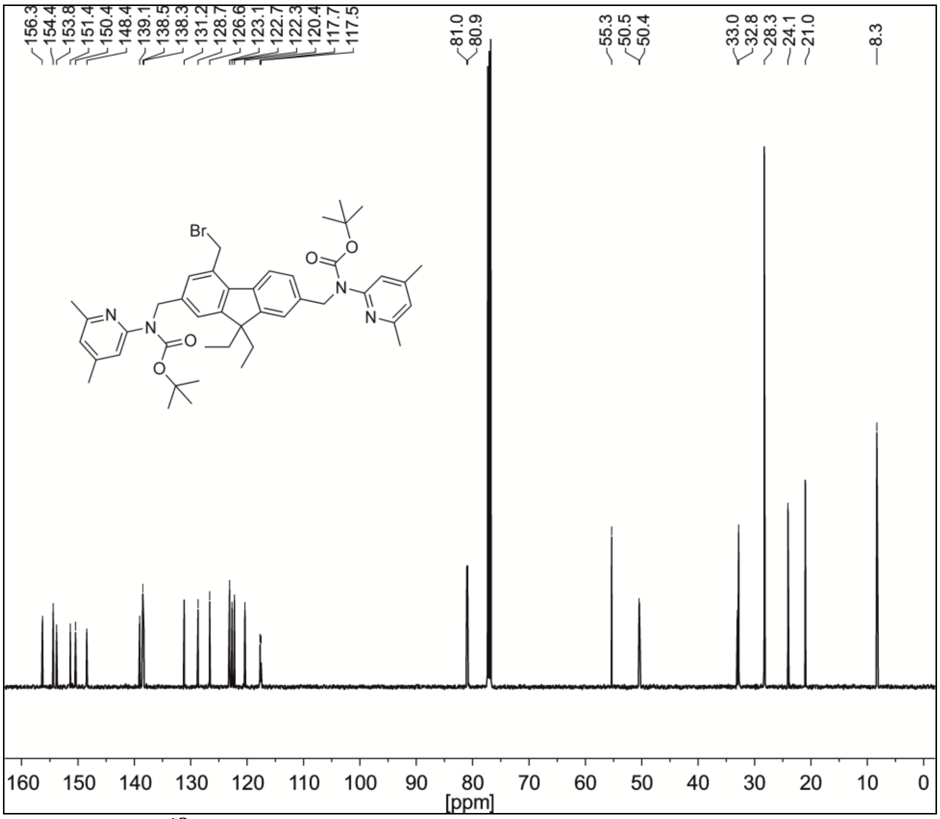

Figure S20. ^{13}C NMR (125 MHz) spectrum of **10** in $CDCl_3$.

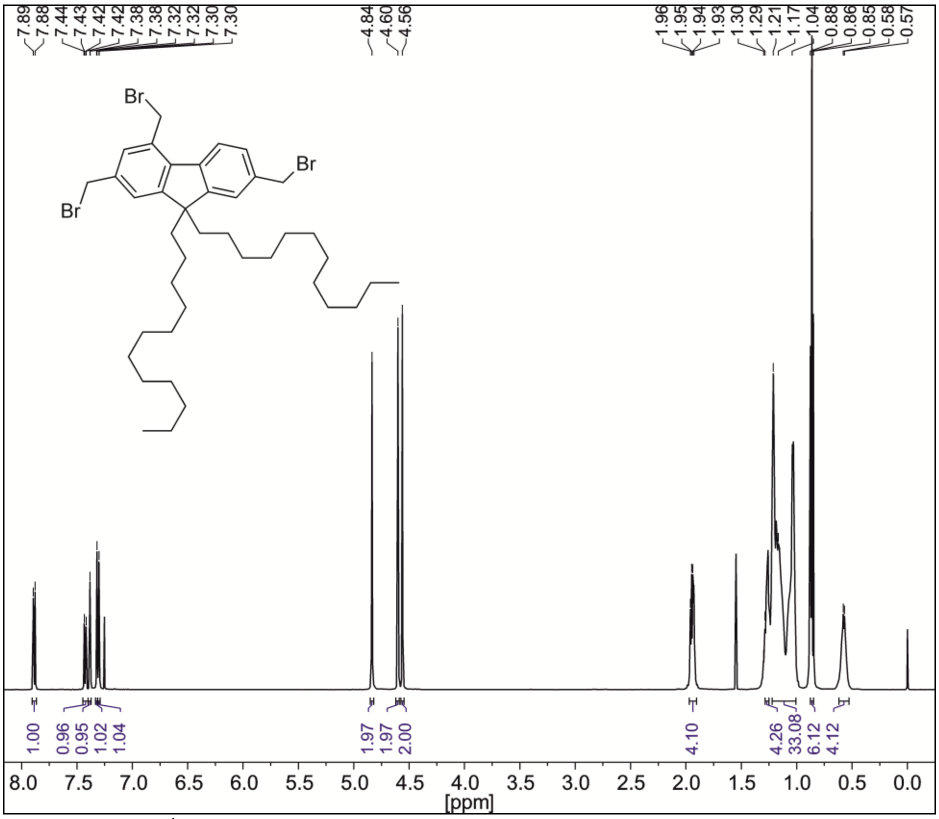

Figure S21. ^{1}H NMR (500 MHz) spectrum of **12** in $CDCl_3$.

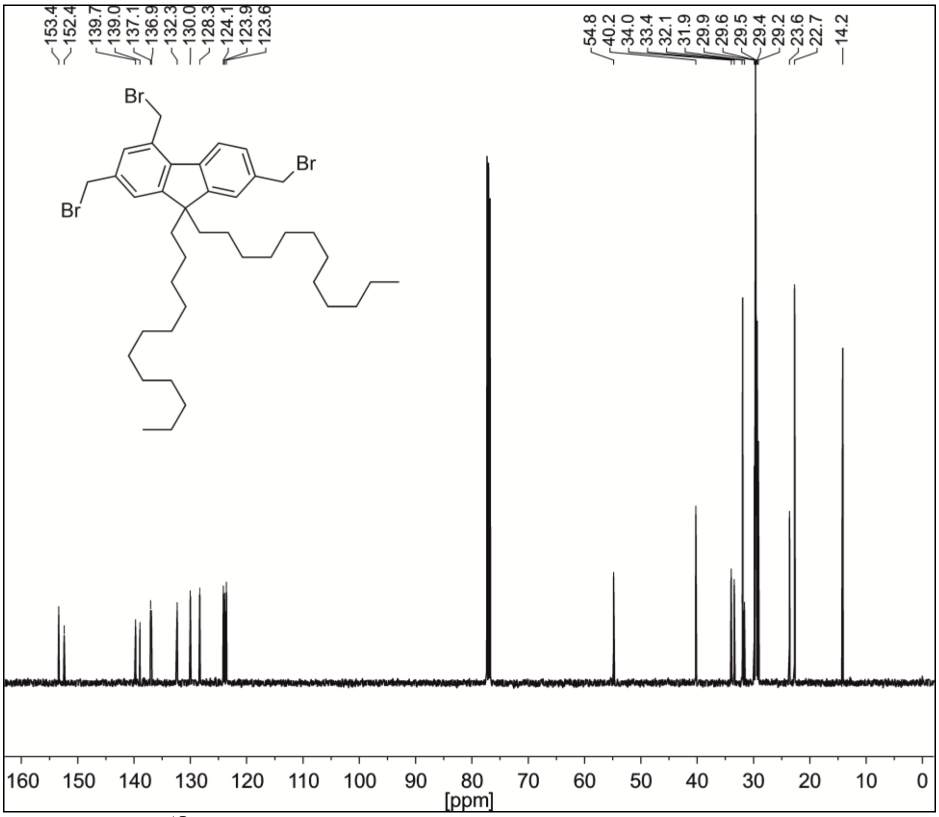

Figure S22. ^{13}C NMR (125 MHz) spectrum of **12** in $CDCl_3$.

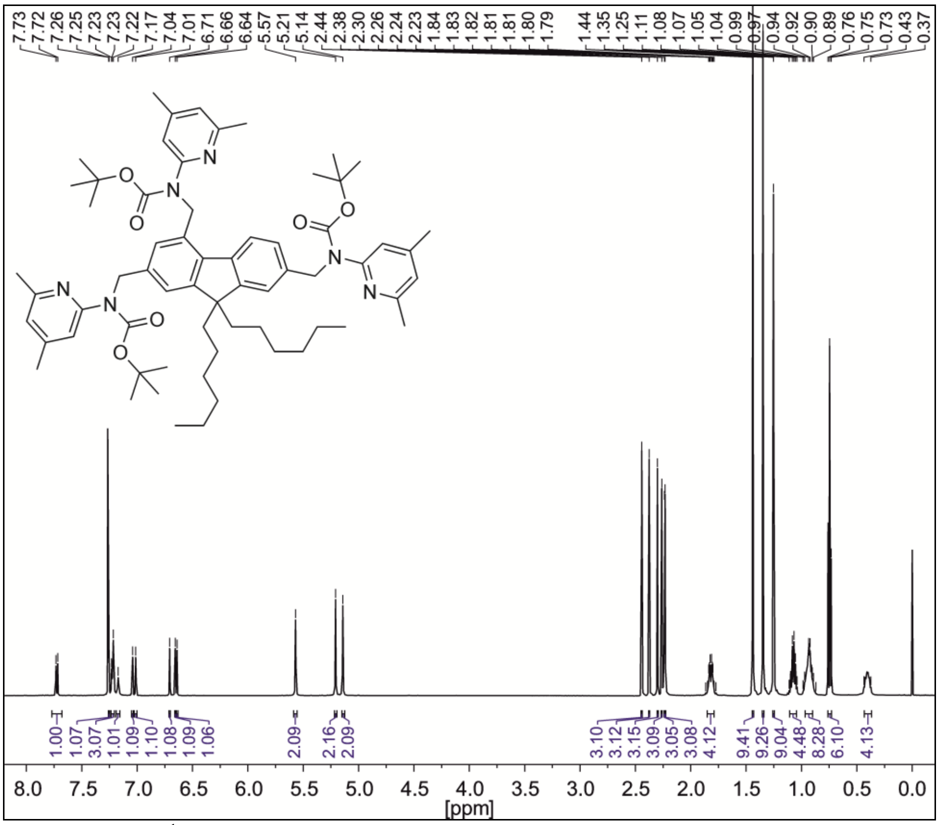

Figure S23. ^{1}H NMR (500 MHz) spectrum of **13** in $CDCl_3$.

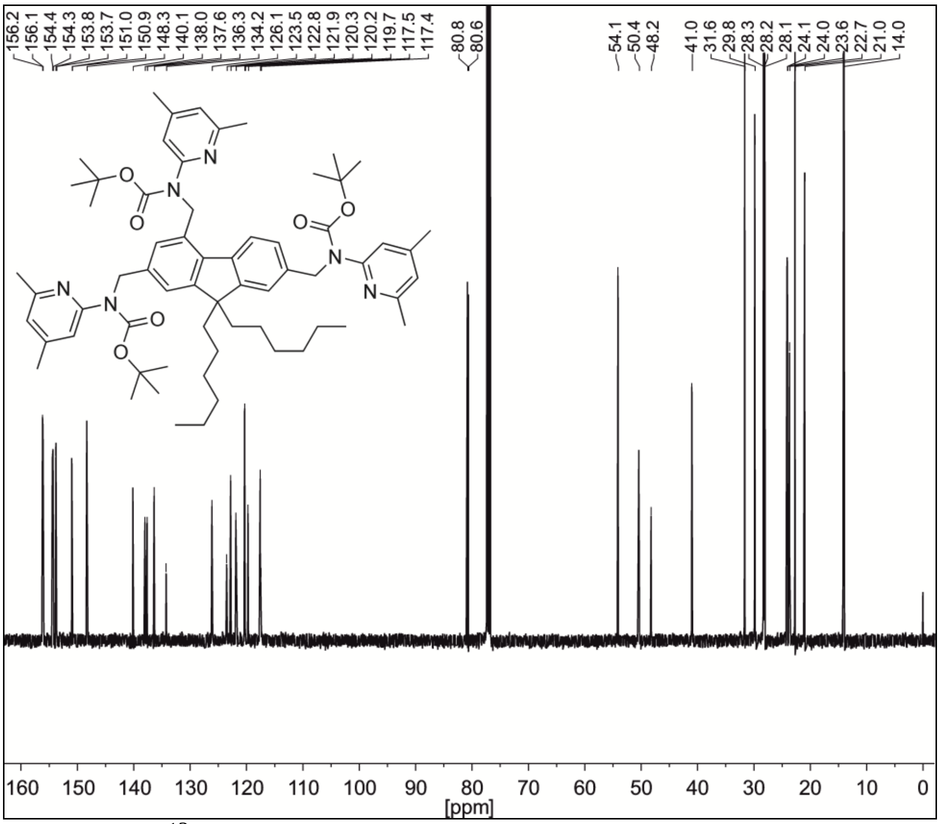

Figure S24. ^{13}C NMR (125 MHz) spectrum of **13** in $CDCl_3$.

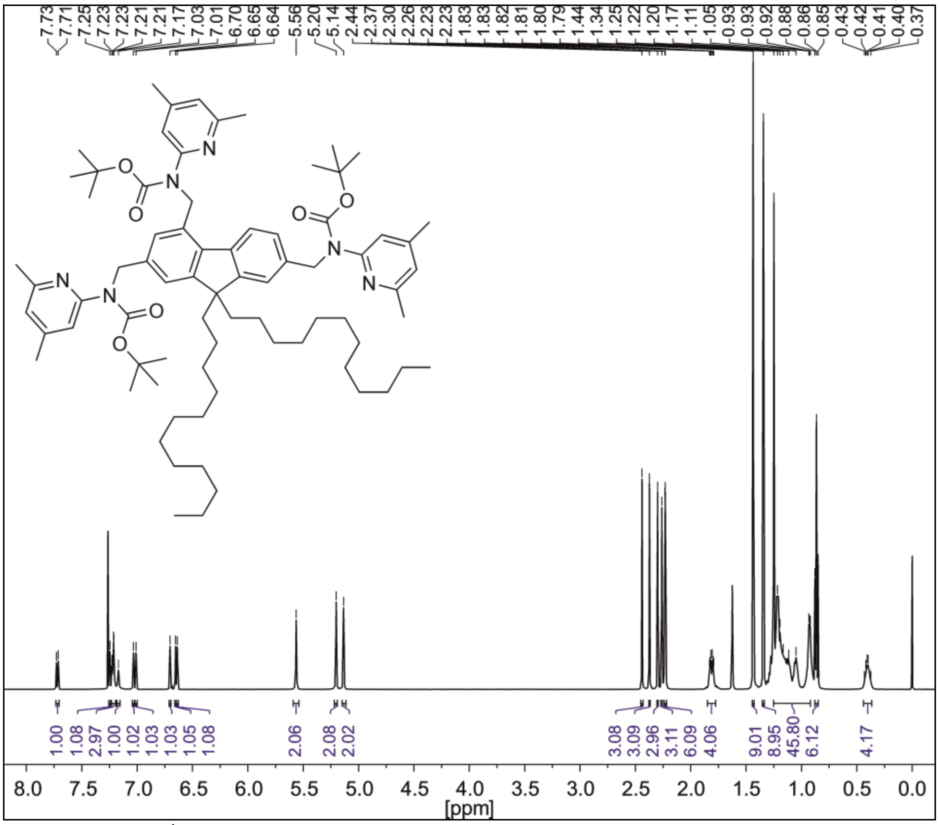

Figure S25. ^{1}H NMR (500 MHz) spectrum of **14** in $CDCl_3$.

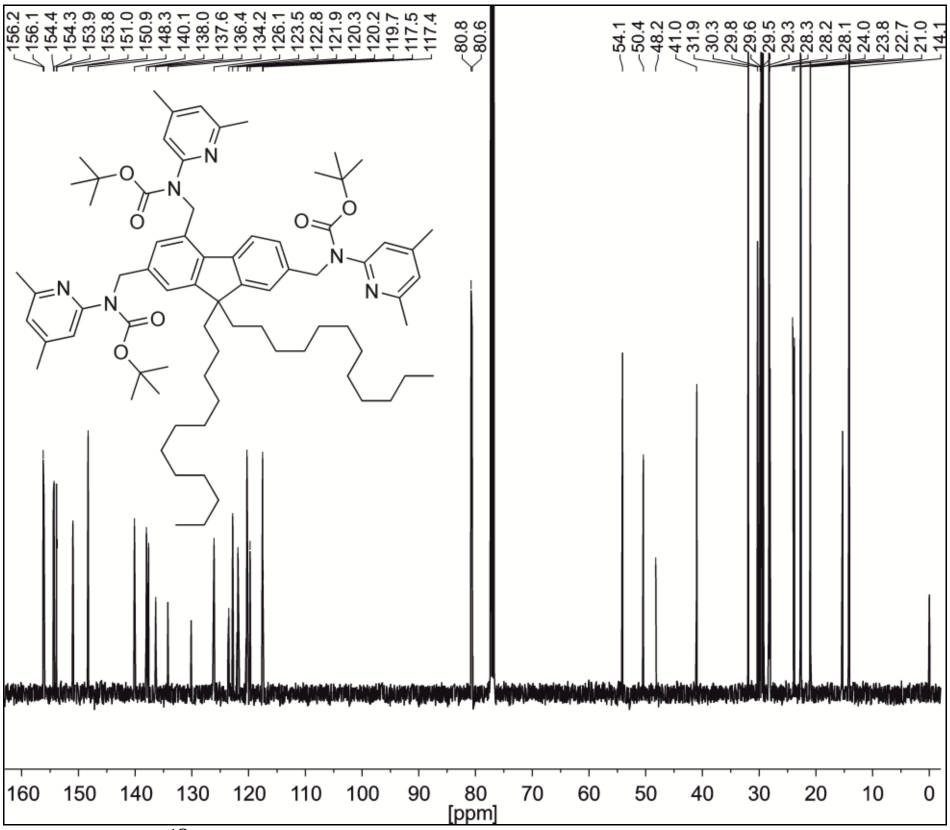

Figure S26. ^{13}C NMR (125 MHz) spectrum of **14** in $CDCl_3$.

7. Description of the performance of ^{1}H NMR and fluorescence titrations

The fluorescence titrations of **1** were carried out in a 50 mL three-necked flask which was connected to a flow cell via an airtight hose system. The samples were transported by a peristaltic pump. The stock solutions of compound **1** were prepared by dissolving **1** and HEPES in methanol, followed by the addition of an equivalent volume of water. 40 mL of the respective solution were placed in the sample flask. The salts were dissolved with another part of this stock solution. From this solution, 5 μL were titrated into the sample flask for each measuring point and the system was equilibrated for one minute. For each fluorescence titration 21 measuring points were recorded (for examples, see Tables S4 and S5).

The fluorescence experiments with compound **3** included 8 samples and were performed using a cuvette (Table S6).

^{1}H NMR titrations were carried out in $CDCl_3$ at 25°C ($CDCl_3$ was stored over activated molecular sieves and deacidified). Stock solutions of **1** and octyl-β-D-glucopyranoside in $CDCl_3$ were prepared. These solutions and $CDCl_3$ were added together in a manner that the concentration of the receptor was kept constant and that of the sugar was varied. At least three ^{1}H NMR titrations were carried out for **1**; for each titration 15 samples were prepared (for an example, see Table S7).

Table S4. Fluorescence titration of receptor **1** ($4 \cdot 10^{-5}$ mol/L) with $InCl_3$ in MeOH/H_2O [1:1 (v/v)].[a]

	$[In^{3+}]$	[Receptor]	Ratio	
	mol/l	mol/l	[Receptor]	$[In^{3+}]$
1	0,00000000	0,00004268	1	0,0000
2	0,00000280	0,00004268	1	0,0657
3	0,00000561	0,00004268	1	0,1314
4	0,00000841	0,00004268	1	0,1971
5	0,00001121	0,00004268	1	0,2627
6	0,00001401	0,00004268	1	0,3283
7	0,00001681	0,00004268	1	0,3940
8	0,00001961	0,00004268	1	0,4596
9	0,00002241	0,00004268	1	0,5251
10	0,00002521	0,00004268	1	0,5907
11	0,00002801	0,00004268	1	0,6563
12	0,00003080	0,00004268	1	0,7218
13	0,00003360	0,00004268	1	0,7873
14	0,00003640	0,00004268	1	0,8528
15	0,00003919	0,00004268	1	0,9183
16	0,00004199	0,00004268	1	0,9838
17	0,00004478	0,00004268	1	1,0493

18	0,00004757	0,00004268	1	1,1147
19	0,00005036	0,00004268	1	1,1801
20	0,00005316	0,00004268	1	1,2456
21	0,00005595	0,00004268		1,3110

[a] Buffered with HEPES (3·10^{-4} mol/l).

Table S5. Fluorescence titration of receptor **1** (1·10^{-6} mol/L) with $InCl_3$ in MeOH/H_2O [1:1 (v/v)].[a]

	[In^{3+}]	[Receptor]	Ratio	
	mol/l	mol/l	[Receptor]	[In^{3+}]
1	0,00000000	0,00000111	1	0,0000
2	0,00000011	0,00000111	1	0,0968
3	0,00000021	0,00000111	1	0,1935
4	0,00000032	0,00000111	1	0,2902
5	0,00000043	0,00000111	1	0,3869
6	0,00000054	0,00000111	1	0,4836
7	0,00000064	0,00000111	1	0,5802
8	0,00000075	0,00000111	1	0,6768
9	0,00000086	0,00000111	1	0,7734
10	0,00000097	0,00000111	1	0,8700
11	0,00000107	0,00000111	1	0,9665
12	0,00000118	0,00000111	1	1,0630
13	0,00000129	0,00000111	1	1,1595
14	0,00000139	0,00000111	1	1,2560
15	0,00000150	0,00000111	1	1,3525
16	0,00000161	0,00000111	1	1,4489
17	0,00000171	0,00000111	1	1,5453
18	0,00000182	0,00000111	1	1,6417
19	0,00000193	0,00000111	1	1,7380
20	0,00000214	0,00000111	1	1,9306
21	0,00000236	0,00000111	1	2,1232

[a] Buffered with HEPES (1·10^{-5} mol/L).

Table S6. Fluorescence measurements with compound **3** and $InCl_3$ in MeOH/H_2O [1:1 (v/v)].[a]

	[In^{3+}]	[Receptor]	Ratio	
	mol/l	mol/l	[Receptor]	[In^{3+}]
1	0,00000000	0,00004602	1	0,0000
2	0,00001394	0,00004602	1	0,3028
3	0,00002787	0,00004602	1	0,6056
4	0,00004181	0,00004602	1	0,9084
5	0,00005574	0,00004602	1	1,2113
6	0,00006968	0,00004602	1	1,5141
7	0,00008361	0,00004602	1	1,8169
8	0,00009755	0,00004602	1	2,1197

[a] Buffered with HEPES (3·10^{-4} mol/L).

Table S7. ^{1}H NMR titration of receptor **1** with octyl β-D-glucopyranoside in $CDCl_3$.

	[Sugar]	[Receptor]	Ratio	
	mol/l	mol/l	[Receptor]	[Sugar]
1	0,00000000	0,00082876	1	0,0000
2	0,00057095	0,00082876	1	0,6889
3	0,00114189	0,00082876	1	1,3778
4	0,00171284	0,00082876	1	2,0668
5	0,00228379	0,00082876	1	2,7557
6	0,00285473	0,00082876	1	3,4446
7	0,00342568	0,00082876	1	4,1335
8	0,00399663	0,00082876	1	4,8224
9	0,00456757	0,00082876	1	5,5114
10	0,00513852	0,00082876	1	6,2003
11	0,00570946	0,00082876	1	6,8892
12	0,00628041	0,00082876	1	7,5781
13	0,00685136	0,00082876	1	8,2670
14	0,00742230	0,00082876	1	8,9559
15	0,00856420	0,00082876	1	10,3338

Screening with metal ions

100 μL of a 10^{-4} M methanolic solution of the corresponding fluorene derivative and 100 μL of a 10^{-4} M aqueous solution of the corresponding metal ion salt were mixed and placed in a well of the microtiter plate.
